# DeepGAMI: deep biologically guided auxiliary learning for multimodal integration and imputation to improve genotype–phenotype prediction

**DOI:** 10.1186/s13073-023-01248-6

**Published:** 2023-10-31

**Authors:** Pramod Bharadwaj Chandrashekar, Sayali Alatkar, Jiebiao Wang, Gabriel E. Hoffman, Chenfeng He, Ting Jin, Saniya Khullar, Jaroslav Bendl, John F. Fullard, Panos Roussos, Daifeng Wang

**Affiliations:** 1 Waisman Center, University of Wisconsin-Madison, Madison, WI 53705 USA; 2 Department of Biostatistics and Medical Informatics, University of Wisconsin-Madison, Madison, WI 53076 USA; 3 Department of Computer Sciences, University of Wisconsin-Madison, Madison, WI 53076 USA; 4 Department of Biostatistics, University of Pittsburgh, Pittsburgh, PA 15261 USA; 5 Center for Disease Neurogenomics, Department of Psychiatry and Department of Genetics and Genomic Sciences, Icahn School of Medicine at Mount Sinai, New York, NY 10029 USA; 6 Mental Illness Research, Education and Clinical Centers, James J. Peters VA Medical Center, Bronx, NY 10468 USA; 7 Center for Dementia Research, Nathan Kline Institute for Psychiatric Research, Orangeburg, NY 10962 USA

**Keywords:** Deep learning, Cross-modality imputation, Auxiliary learning, Genotype–phenotype prediction, Cell-type gene regulatory networks, Schizophrenia, Alzheimer’s disease, Prioritizing disease risk variants

## Abstract

**Background:**

Genotypes are strongly associated with disease phenotypes, particularly in brain disorders. However, the molecular and cellular mechanisms behind this association remain elusive. With emerging multimodal data for these mechanisms, machine learning methods can be applied for phenotype prediction at different scales, but due to the black-box nature of machine learning, integrating these modalities and interpreting biological mechanisms can be challenging. Additionally, the partial availability of these multimodal data presents a challenge in developing these predictive models.

**Method:**

To address these challenges, we developed DeepGAMI, an interpretable neural network model to improve genotype–phenotype prediction from multimodal data. DeepGAMI leverages functional genomic information, such as eQTLs and gene regulation, to guide neural network connections. Additionally, it includes an auxiliary learning layer for cross-modal imputation allowing the imputation of latent features of missing modalities and thus predicting phenotypes from a single modality. Finally, DeepGAMI uses integrated gradient to prioritize multimodal features for various phenotypes.

**Results:**

We applied DeepGAMI to several multimodal datasets including genotype and bulk and cell-type gene expression data in brain diseases, and gene expression and electrophysiology data of mouse neuronal cells. Using cross-validation and independent validation, DeepGAMI outperformed existing methods for classifying disease types, and cellular and clinical phenotypes, even using single modalities (e.g., AUC score of 0.79 for Schizophrenia and 0.73 for cognitive impairment in Alzheimer’s disease).

**Conclusion:**

We demonstrated that DeepGAMI improves phenotype prediction and prioritizes phenotypic features and networks in multiple multimodal datasets in complex brains and brain diseases. Also, it prioritized disease-associated variants, genes, and regulatory networks linked to different phenotypes, providing novel insights into the interpretation of gene regulatory mechanisms. DeepGAMI is open-source and available for general use.

**Supplementary Information:**

The online version contains supplementary material available at 10.1186/s13073-023-01248-6.

## Background

The genotype–phenotype association has been found in many biological systems, such as brain-related diseases and behavioral traits. This association is very important as it will help us understand underlying cellular and molecular mechanisms like genes and pathways that causally affect the phenotypes [1, 2]. Many genome-wide association studies (GWAS) determine the association of genetic variants with many heritable diseases [3, 4], including neurodegenerative and psychiatric diseases like Alzheimer’s disease (AD) [5–8] and schizophrenia (SCZ) [9, 10]. Despite the ground-breaking findings from these GWAS studies, they have some limitations. Firstly, association studies do not imply causation and require further downstream analysis and validations. Secondly, GWAS studies are independent studies that try to find the relationship between variants and disease individually and ignore the combined effect. Finally, the SNPs having small effect sizes go undetected as they do not meet the threshold criteria of the existing studies [11]. There have been several computational attempts outside the GWAS studies to discover genotype–phenotype association. Most of these attempts involve regression [12, 13]. Polygenic Risk Scores (PRS) [14] is the widely used method that looks at the linear combined effect of several variants on the phenotype. Modern machine learning techniques have been applied to predict the functionality of these phenotypes. For example, Zhang et al. [15] grouped several SNPs based on LD structure and used it as an input to the CNN model to predict AD progression. However, predicting phenotypes from genotypes remains challenging, primarily due to complex underlying molecular and cellular mechanisms. These methods perform genotype-to-phenotype prediction without considering various underlying intermediate phenotypes and mechanisms like gene expression and epigenome regulation.

For a mechanistic understanding from genotype to phenotype, several studies have shown that these variants influence disease risks by altering cell-type regulatory elements that affect the underlying gene expressions, which in-turn affect the disease phenotype [16, 17]. This resulted in studying the effects of genotypes on gene expression. Expression quantitative trait loci (eQTL) studies focus on associating genetic variants to gene expression instead of disease phenotypes [18–23]. They have proved to be a critical step in investigating gene regulation and have identified numerous eQTLs modulating the expression of disease genes. Transcriptome-wide association studies (TWAS) aim at identifying gene-trait interaction by combining GWAS and gene expression. The effect of genetic variation on gene expression is first studied, and then these expression profiles are statistically associated with the traits [24–31]. PrediXcan [32] is another approach imputing gene expression from eQTLs and mapping trait-associated loci based on the imputed gene expression data. A possible drawback in such association studies is that co-expressed gene patterns often lead to prioritizing non-causal genes [33].

It is also necessary to analyze genes and other regulatory elements that impact disease phenotypes. Several methods have attempted to associate genes with disease risks using gene expression profiles directly. For example, one study collected gene expression profiles from three publicly available AD datasets to predict the onset of AD disease using a variational autoencoder [34]. DeepWAS [35] predicted epigenomic functions of the genetic variants using DeepSEA [36] and then applied regression to predict the phenotype. A different approach is using gene regulatory networks (GRNs). GRNs represent a group of genes and various regulatory elements working together to control the expression of other genes and are cell-type specific. They facilitate understanding of various cell operations which allows better understanding of disease initiation and progression [37]. They have proven helpful in mapping molecular interactions [38, 39] and biomarkers for brain diseases [40–42]. However, a major drawback of these methods is that they consider each omics individually and thus miss the relationship between multiple omics.

As biological processes involve a complex interaction with multi-omics, emerging multimodal data enables studying such mechanisms at different scales. Several studies have attempted to integrate multi-omics data for brain disease predictions like SCZ [43–45] and AD [46–48]. Some studies have used known biological findings (e.g., GRNs, eQTLs) to guide feature selection or integrate several omics for disease prediction. For example, Wang et al. [49] used a deep Boltzmann machine (DBM) with GRNs guiding the internal connections to improve predicting neuropsychiatric diseases. Varmole [50] integrated gene expression and genotype (SNPs) using a deep neural network architecture where GRN and eQTLs guide the relationship between the input and the first hidden layer. While most studies consider disease outcomes (case versus control) as the phenotypes, more complex phenotypes, e.g., neuropathological, and cognitive phenotypes for AD, remain understudied. Studying the genetic effects of those disease phenotypes has great potential to deeper understand underlying cellular, molecular, and pathological mechanisms. Also, GRNs and eQTLs provide functional genomic relationship information. Most existing machine learning methods use raw -omics data as inputs but cannot handle these relationship data. Recent advancements in graph learning have led to the use of graph neural networks (GNNs) for integrating multi-omics data and use relationships like MOGONET [51] and moGCN [52]. The relationship data is converted into graphs and given to GNNs as inputs. GNNs apply different neural network methods on the graphs to extract useful latent features which are then used for various supervised and unsupervised tasks. However, these methods mainly focus on similarity networks (like patient similarity networks) and are not suitable for functional genomic networks as these networks are directed, sometimes bipartite (e.g., eQTL networks) and relationship topology is complicated.

Another challenge for genotype–phenotype studies is multimodal data integration. Due to several factors (e.g., experimental costs, sample availability), multi-omics data will often be partially available for individuals [53], e.g., available genotypes versus limited gene expression, chromatin, or imaging data [54, 55]. Including partially available multimodal datasets in a predictive model will be challenging due to the lack of matched samples and missing modalities. This calls for cross-modal imputation. Cross-modal imputation involves estimating data modalities from available multiple modalities. This will help us infer missing modalities and aid in phenotype predictions. Several computational methods have been developed for cross-modal inference. MOFA [56] uses Bayesian approach for cross-modality estimation by projecting the modalities onto a low-dimensional space. BABEL [57] trains a joint autoencoder on paired multimodal data to impute one modality from another by minimizing a reconstruction and cross-modality loss. scVAEIT [58] proposed a method for mosaic integration that uses a masking procedure to learn a joint representation of cells sequenced across technologies and the distribution of missing modalities. scJoint [59] integrates scRNA-seq and scATAC-seq data using cell-type label information from scRNA-seq through transfer learning and embedding the annotated cells in a lower-dimensional space. There are no existing methods that perform cross-modal imputation alongside predicting disease phenotypes to the best of our knowledge.

Auxiliary learning is a type of learning technique aimed at improving the generalization of the primary task by learning secondary tasks along with the primary task [60–63]. The secondary task also called the auxiliary task is a subtask to be trained along with the primary task where the features are shared between the tasks resulting in additional relevant feature extraction useful for the primary task, and thus is usually defined in terms of estimating entities relevant to solving the primary task [64]. Implementing auxiliary learning involves adding supplementary cost functions to the primary cost of the neural network model [65]. Auxiliary learning has been very successful in reinforcement learning [60, 66, 67], computer vision [62, 68, 69], and autonomous driving assistance [70, 71]. Recently, it has been used in the biomedical domain with applications in screening skin cancer from microscopy images [72], and covid-19 detection from CT images [73]. Although auxiliary learning has not been applied for imputing multimodal data for genotype–phenotype prediction, the closest approach is SCENA [74] which estimates the gene–gene correlation matrix using ensemble learning and auxiliary information for single-cell RNA-seq (scRNA-seq) data where the auxiliary information is used in the form of gene networks and other relevant RNA-seq data. Similarly, DeepDiff [75] predicts cell-type-specific differential gene expression from epigenetics by using cell-type gene expression predictions as auxiliary tasks.

In summary, genotype–phenotype predictions are very important in understanding molecular and cellular mechanisms, but existing genotype–phenotype methods have the following limitations: (I) Statistical methods such as Polygenic Risk Score (PRS) predict phenotypes directly from genotype. They are mostly linear models that cannot tackle genomic variants’ nonlinear effects and involve association studies that predict the correlation between genotype and phenotype but cannot explain how the inherited mutations are associated with the phenotype [76, 77]. Moreover, these methods do not consider intermediate phenotypes like molecular activities that significantly contribute to phenotypes; (II) Emerging multi-omics data at the population level enables machine learning which studies such mechanisms at different scales from genotype to phenotype. However, due to the black-box nature of many machine learning techniques, it is challenging to integrate these multiple modalities and interpret the biological mechanisms after prediction, especially when some modality is missing; (III) Functional genomic relationships like GRNs and eQTLs guide us in understanding these molecular mechanisms. However, most existing machine learning methods, including GNNs, cannot handle this kind of relationship data as they do not have a spatial relationship like graphs, and significant effort is required to convert them into a graph-like structure. (IV) Several methods focus on cross-modality estimation for single-cell multi-omics data (e.g., MOFA [56], MultiVI [78], Polarbear [79]) but not in the realm of disease types and clinical phenotypes.

To address these limitations, we propose DeepGAMI: Deep biologically guided auxiliary learning for multimodal integration and imputation to improve phenotype prediction. DeepGAMI is a novel deep learning model that enables cross-modal imputation, predicts clinical phenotypes, and prioritizes phenotypic tissue- or cell-type functional genomics. Our contributions are three-fold. Firstly, DeepGAMI provides a biologically guided neural network framework for genotype–phenotype prediction using biology-guided dropconnect [80]. It integrates genotype and gene expression data guided by prior biological knowledge of QTLs and GRNs. Secondly, it introduces an auxiliary learning layer that performs cross-modal estimation by learning relationships between modalities. This enables the model to take a single modality and use the estimated values of the other modality for disease prediction. Thirdly, DeepGAMI deciphers the black-box nature of the neural networks to prioritize genes and SNPs contributing to disease phenotypes. We applied DeepGAMI to multiple emerging multimodal datasets, including population-level genotype and bulk and cell-type gene expression data for SCZ cohort [49], genotype and gene expression data for AD cohort [55], and single-cell multimodal data comprising transcriptomics and electrophysiology for neurons in the mouse visual cortex [81]. We found that DeepGAMI outperforms existing methods in predicting phenotypes among these datasets and prioritizes genes, SNPs, and electrophysiological features showing biological interpretability. DeepGAMI is open-source and available at https://github.com/daifengwanglab/DeepGAMI.

## Methods and materials

### DeepGAMI overview

**Fig. 1:**
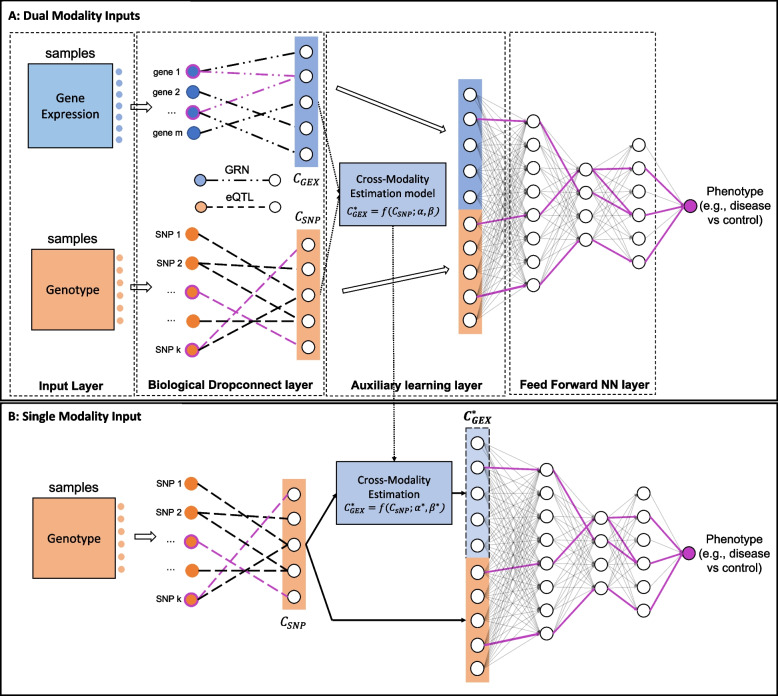
Architecture of DeepGAMI. **A** DeepGAMI first uses available multimodal features for training the predictive model, e.g., SNP genotypes (orange) and gene expression (blue) of individuals from the major applications in this study. In particular, it learns the latent space of each modality (e.g., consisting of latent features at the first transparent hidden layer). This learning step is also regularized by prior knowledge enabling biological interpretability after prediction, i.e., the input and latent features are connected by biological networks (biological DropConnect). For instance, the input transcription factor genes can be connected to target genes as their latent features (e.g., *C*_*GEX*_) by a gene regulatory network (GRN). The input SNPs can be connected to associated genes as their latent features (e.g., *C*_*SNP*_) by Expression quantitative trait loci (eQTLs). Notably, an auxiliary learning layer is used to infer the latent space of one modality to another, i.e., cross-modality imputation. For instance, DeepGAMI learns a transfer function *f*(.) to estimate *C*_*GEX*_ from *C*_*SNP*_. Finally, the latent features are concatenated and fed to the feed-forward neural networks for phenotype predictions, e.g., classifying disease vs. control individuals. **B** Using the learned predictive model from multimodal input along, DeepGAMI can predict phenotypes from a single modality, e.g., SNP genotypes of new individuals. Specifically, it first imputes other modal latent spaces using the optimal transfer function f^*^(.) and then feeds both imputed and input latent features into downstream neural network predictions, e.g., C^*^_GEX_ from *C*_*SNP*_

DeepGAMI is a multi-view deep learning model that integrates multimodal data for predicting phenotypes (Fig. 1, Methods and materials). Importantly, it uses auxiliary learning to learn the latent space of one modality from another, thereby enabling us to predict phenotypes from the available modalities by cross-modality imputation. We denote the primary task as ${f}_{\theta }^{pri}\left({X}_{1},{X}_{2}\right)$ that takes available multimodal inputs ${X}_{1}$ and ${X}_{2}$ with parameters $\theta$ to predict phenotypes, e.g., ${X}_{1}$ and ${X}_{2}$ can be SNP genotypes and gene expression levels of individuals. The auxiliary task is denoted by ${f}_{\theta }^{aux}\left({X}_{1},{X}_{2}\right)$ to learn ${C}_{{X}_{2}}$(latent space of input ${X}_{2}$) from ${C}_{{X}_{1}}$(latent space of input ${X}_{1}$). Using the trained model with auxiliary task, DeepGAMI can predict the phenotypes of samples with a missing modality. Specifically, it first imputes other modal latent spaces using the auxiliary learning function and then feeds both imputed and input latent spaces into the primary task for phenotype prediction. DeepGAMI uses feed-forward neural networks as it incorporates biological knowledge in terms of directed functional relationship networks like GRNs and eQTLs which aid in deciphering the black box and help us prioritize genes, SNPs, and other biological features for phenotypes. A list of all hyperparameters with its search space is available in Additional file 2: Table S1 and the total number of trainable parameters of DeepGAMI for each dataset is shown in Additional file 2: Table S2.

We compared the classification performance of DeepGAMI with three traditional classifiers: (1) Random Forest classifier (RF), (2) Support Vector Machine SVM), (3) Fully connected neural network classifier (MLP), and four state-of-the-art methods: (4) Logistic Regression (LR) [12], (5) Lasso Regression (Lasso) [13], (6) Varmole [50], (7) Mogonet [51], on several multi-omics datasets. RF, NB, and MLP were trained on the concatenation of multi-omics data.

### Model design

The DeepGAMI model consists of four main layers.

#### Input layer

The input layer consists of two data modalities, e.g., gene expression and SNP genotypes. Each row of the input matrix represents feature vector of a sample. For example, the gene expression matrix contains gene expression profiles of *K* samples and *n* TFs and is represented by ${X}^{GEX} \in {R}^{K*n}$. Similarly, the genotype matrix consists of dosage information of *K *samples and *l* SNPs, ${X}^{SNP}\in {R}^{K*l}$.

#### Biological DropConnect layer

DropConnect is a regularization mechanism that sets random activation units to zero in each layer. It differs from dropout, which sets the random output units to zero while the former sets the connection weights to zero [80]. For our purpose, instead of randomly setting activations to zero, we guide the activations using prior biological knowledge, as shown in Eqs. 1 and 2.

1 $${C}^{SNP}_{k}= \sigma \left({X}^{SNP}_{k}\left({w}_{1}\odot {m}^{eQTL}\right)+{b}_{1}\right)$$

and

2 $$C_k^{GEX}=\sigma(X_k^{GEX}\left(w_2\odot m^{GRN}\right)+b_2),$$

where $${C}^{SNP}_{k}\ and\ {C}^{GEX}_{k}$$ represent intermediate layer with nodes for *k*^*th*^ sample, $${X}^{GEX}_{k}\ and\ {X}^{SNP}_k$$  are the *k*^*th*^ column of *X*^*GEX*^ and *X*^*SNP*^ and represent gene expression and dosage data for *k*^*th*^ sample, *w*_*1*_ and *w*_*2*_ are weight matrices with dimension *R*^l*p^ and *R*^n*p^ respectively,* b1* and *b2* are the bias vectors of length *p*, and $$\odot$$ is the Hadamard product (element-wise multiplication). We do not encode self-loops and inter-connections among the input features (TFs, SNPs, etc.). If there are multiple levels of connections (e.g., $${S}_{1}\to {S}_{2}\to {T}_{1}$$), we treat them as two separate connections ($${S}_{1}\to {S}_{2}$$ and $${S}_{2}\to {T}_{1}$$). In such cases, the genes ($${S}_{2}$$ from the above example) appear under both the input layer and the intermediate layer. The biological DropConnect is applied separately for genotype and gene expression. Masking filter ($$m)$$ encodes biological drop connections (Eqs. 3 and 4).

3 $${m}_{\left\{i,j\right\}}^{eQTL}= \left\{\begin{array}{c}1\ if\ SNP\ i\ is\ associated\ with\ gene\ j\\ 0\ othewise\end{array}\right.$$

4 $$m_{\left\{i,j\right\}}^{GRN}=\left\{\begin{array}{c}1\;if\;TF\;i\;regulates\;gene\;j\\0\;othewise\end{array}\right.,$$

where *m*^*eQTL*^ ∈ *R*^l*p^ and *m*^*GRN*^ ∈ *R*^n*p^. The underlying idea of this layer is to model the regulatory relationships among genes such as TFs to genes and SNPs to genes. The input $${X}^{GEX}$$ represents all TFs as features and the intermediate layer nodes ($${C}^{GEX}$$) which represent genes for all samples, are the output of the biological DropConnect layer. The connections between these TFs and genes are established using GRNs ($${m}^{GRN})$$. Similarly, the connections between SNPs and genes are established using eQTLs ($${m}^{eQTL}$$). The model is trained to learn the weights for these connections. This will help us prioritize important features (SNPs, genes, enhancers, etc.) and important interactions (SNP-gene and gene–gene) contributing to the phenotype. The output of this layer is referred to as the latent space of the input matrix.

#### Auxiliary learning layer

Each data modality from the input layer goes through the biological DropConnect layer producing a set of output nodes of equal dimension (${C}^{GEX}, {C}^{SNP}$). This layer aims to learn the latent space of one modality from the other. We consider a linear relationship between the two latent spaces, computed using Eq. 5.

5 $${{C}^{{GEX}^\star} = f}_{\theta }^{aux}\left({C}^{SNP}\right)=\alpha{C}^{SNP}+ \beta,$$

where $\alpha\ and\ \beta$ are scalar units representing weight and bias. We then concatenate the two latent space vectors and send them to a feed-forward neural network. One can get an average signal of the latent space vectors, but we decide not to pursue it as each latent node can be activated from either both the inputs or only one input.

#### Feed-forward classification layer

The concatenated gene layer is given to a fully connected feed-forward neural network with multiple hidden layers where each neuron in the hidden layers receives inputs from all previous layer outputs. ReLU activation is applied that forces negative weights to zero and handles non-linearity. ReLU activation is defined as shown in Eq. 6.

6 $$ReLU\left(X\right)=\mathrm{max}\ (0, X)$$

The final hidden layer is given to a softmax function to predict the output classes from the input provided by Eq. 7.

7 $$softmax\left({z}_{i}\right)= \frac{{e}^{{z}_{i}}}{{\sum }_{j}{e}^{{z}_{j}}}$$

where $z$ represents the neuron values from the previous layer.

### Training of DeepGAMI model

We split the input data into training (80%) and testing (20%) sets and performed fivefold cross-validation (CV) on the training set for feature selection and identifying the optimal parameter combination. We then pick the best performing model based on the five-fold CV and evaluate the final performance on the test set. Training DeepGAMI model involves minimizing the overall loss function which is a combination of primary task (phenotype prediction) loss and secondary task (cross-modality estimation) loss. The loss function used for the primary task is the cross-entropy loss (Eq. 8) and mean squared error (MSE) loss is used for the secondary task (Eq. 9).

8 $${\mathcal{L}}^{pri}\left(y, \widehat{y}\right)=\mathcal{L}\left( {f}_{\theta }^{pri}\left({X}^{SNP}, {X}^{GEX}\right), y\right)= -\frac{1}{K} {\sum }_{k=1}^{K}{y}_{k}\mathrm{log}({\widehat{y}}_{k})$$

9 $${\mathcal{L}}^{aux}\left({C}^{GEX}, {C}^{SNP}\right)= \frac{1}{K} {\sum }_{k=1}^{K}{\left({C}^{GEX}_{k}- {f}_{\theta }^{aux}\left({C}^{SNP}_{k}\right)\right)}^{2}$$

The overall objective loss function for the model is computed using Eq. 10.

10 $$\mathit{arg}\underset{\theta }{\mathrm{min}} ( {\mathcal{L}}^{pri}+ \lambda{\mathcal{L}}^{aux})$$

where $i$ represents the ${i}^{th}$ training sample, $\theta$ is a set of parameters. $y\ and\ \widehat{y}$ represents the ground truth and the predicted labels respectively.

#### Evaluation metrics

As traditional accuracy measure can be misleading on skewed datasets, we used balanced accuracy (BACC, Eq. 11) and area under the receiver operating characteristic curve (AUC) to evaluate the performance of our model and other baseline comparison models.

11 $$BACC=\frac{sensitivity+specificity}{2}$$

$$sensitivity=\frac{TP}{TP+FN}$$

$$specificity=\frac{TN}{TN+FP}$$

where TP represents true positive, FP represents false positive, TN represents true negative, and FN is false negative. While the loss function includes both primary and auxiliary task losses, the performance of the model is based on BACC and AUC computed on the primary task.

#### Hyperparameter tuning for training DeepGAMI

DeepGAMI is trained using Adam Optimizer [82] with default parameters of ${\beta }_{1}=0.9$ and ${\beta }_{2}=0.999$. The model runs for a maximum of 100 epochs with early stopping enabled to avoid overfitting. Tuning of several hyperparameters (like number of latent dimensions, number of hidden layers in the feed forward network, dropout rate) is required as it has a huge impact on the prediction performance. All hyperparameters with the search space implemented in DeepGAMI model are present in Additional file 2: Table S1. The optimal hyperparameter combination was selected using a grid search based on the fivefold CV results on the training set. DeepGAMI is coded in python using Pytorch [83] library.

### Feature prioritization

Integrated gradient (IG) [84] is a widely used technique for feature prioritization, IG attributes the model’s prediction for its input features by computing gradients for each input and measures the change in the output response based on the small changes in the input using Eq. 12. IG is implemented using Captum [85] package in python.

12 $$I{G}_{i}\left(x\right)=\left({x}_{i}-{x}_{i}^{\prime}\right){\int }_{\alpha =0}^{1}\frac{\partial F\left({x}^{\prime}+ \alpha \left(x-{x}^{\prime}\right)\right)}{\partial {x}_{i} } \partial \alpha$$

where $i$ is the ${i}^{th}$ feature, $x$ and ${x}^{\prime}$ are input and baseline, and $\frac{\partial F(x)}{\partial {x}_{i}}$ is the gradient of $F(x)$ along the ${i}^{th}$ feature.

IG provides feature importance scores where a higher score indicates higher importance of the feature. We first applied IG on the trained model (model with the best performance on the training set with the optimal hyperparameter setting) for generating feature importance scores for the input nodes (SNPs, TFs) and latent space nodes (genes) for the test samples. This will help us identify important SNPs and TFs attributed to the phenotype’s outcomes. We then applied IG on the same model to extract link importance scores for the test samples. The link importance score gives us the importance score for the links between the input and the intermediate layer (Biological DropConnect layer). This will provide potential clues in understanding the underlying relationships (SNPs to genes and TFs to genes) for the given phenotype. Using this link importance score, we can fine-tune prioritized regulatory networks for phenotypes.

### Enrichment analysis

From the prioritized functional links between SNPs and TFs to genes, we extract the genes having the most important links (top 10% of the link importance scores). We then perform enrichment analysis on these genes using Metascape [86]. The enrichments with binomial FDR *p*-value < 0.05 were reported in the prioritized genes.

### Multimodal datasets and processing

#### Data preprocessing

The datasets vary between different cohort as they are extracted in different platforms using different protocols. We use the same preprocessing pipeline to process these inputs to the deep learning model and to reduce the effect of curse of dimensionality. We first use Student’s *t* test (for binary phenotypes) and ANOVA (for multi-class phenotypes) for feature selection on the training set. As our intermediate layer consists of genes, we filter and keep the common genes between the two biological networks (e.g., GRNs, eQTLs). We only keep selected features (SNPs, TFs) present in these two networks. Next, we applied StandardScaler() function from scikit-learn [87] for the two modalities separately which scales the data such that they have zero mean and unit variance. StandardScaler() is computed using the following equation:

13 $$z=\frac{(x-\mu )}{\sigma }$$

where $x$ is the input, $\mu$ is the mean, and $\sigma$ is the standard deviation. We also provide the users an option to choose the standardization of their choice (currently DeepGAMI supports minmax, log, and standard normalization).

#### Schizophrenia

We used the population-level bulk RNA-seq and genotype data for the human dorsolateral prefrontal cortex (DLPFC) from PsychENCODE [49] for predicting SCZ versus healthy individuals. RNA-seq data consists of normalized gene expression of 14,906 genes for 1818 individuals. We extracted 146,763 eQTLs from GTEx consortium [88] for the human brain frontal cortex (BA9), and used GRNs from the PsychENCODE consortium. We first use Student’s *t* test for feature selection (keep significant SNPs and genes). We do not consider LD structure of the SNPs. We then include SNPs and genes which are present in eQTLs and GRN. Based on this pipeline (see Input Data Preprocessing), we ended up with 2080 SNPs, 126 TFs, and 84 intermediate layer genes as features.

We also tested DeepGAMI on the genotype and cell-type specific gene expression data from the CommonMind Consortium imputed using bMIND [89] and a reference panel of 4 cell populations: GABAergic (i.e., inhibitory) neurons, glutamatergic (i.e., excitatory) neurons, oligodendrocytes, and a remaining group composed mainly of microglia and astrocytes. The reference panel for each cell population was constructed by taking the mean log2 counts per million for each gene across 32 brain donors [90]. With the prior information from this reference panel, bMIND adopts a Bayesian approach to impute the cell-type-specific expression of each gene in each bulk sample from gene expression assayed from brain homogenate. We used cell-type-specific eQTLs [23] and applied scGRNom [91] to predict cell-type GRNs.

#### Alzheimer’s disease

We used the bulk RNA-seq data in DLPFC and genotype data from the ROSMAP cohort [55] for our analysis in Alzheimer’s disease. We used preprocessed bulk RNA-seq data (quantile normalized and batch effect removed) which contains the FPKM gene expression values.

For genotype data, we extracted SNP array (generated using Affymetrix GeneChip 6.0) dosage information for 1709 individuals. We extracted 146,763 eQTLs from GTEx consortium [88] for the human brain frontal cortex (BA9), and used GRNs from the PsychENCODE consortium [49]. Clinical phenotypes include cognitive diagnosis (COGDX) score ranging between 0 and 6, CERAD score (semi-quantitative measurement of the neuritic plaques useful for determining AD) ranged 0–4, and BRAAK score (semi-quantitative measurement for neurofibrillary tangle pathology) containing six stages. We coded the BRAAK phenotype into two classes (early-stage AD which contains BRAAK stages of 0–3 and late-stage AD containing BRAAK stages of 4–6), CERAD scores into three classes (No AD with scores 3–4, AD probable with score 2, and AD definite with score 0–1), and COGDX into three categories (No cognitive impairment (CI) with scores 0–1, mild CI with scores 2–3, and CI(AD/Dementia) with scores 4–6) using the coding available in ROSMAP. We used analysis of variance test (ANOVA) to filter out SNPs and genes with high variance except for BRAAK, where we used a *t*-test instead. We then intersected the SNPs and genes with eQTLs and GRN. We ended up with 229 early-stage AD individuals and 275 late-stage AD individuals for the BRAAK score phenotype. For the COGDX phenotype, we had no CI (*n* = 166), mild CI (*n* = 130), and CI (AD/Dementia, *n* = 208) individuals. Finally, for the CERAD phenotype, we ended up with no AD (*n* = 184), AD probable (*n* = 171), and AD definite (*n* = 149) individuals.

#### Mouse visual cortex

Patch-seq dataset includes transcriptomics and electrophysiological (ephys) data for 4435 neuronal cells in mouse visual cortex [81]. We used the cell cortical layers (cell location in the visual cortex) as the cellular phenotype: L1, L2/3, L4, L5, L6. We followed the data extraction and preprocessing in DeepManReg [92] and ended up with 41 ephys features and 1000 genes for 3654 cells. We also used 112 layer4 (L4) neuronal cells Patch-seq data for independent testing [93]. For this application, the inputs contain the gene expression data ${X}^{GEX} \in {R}^{K*n}$ of *n* genes and *K* samples, and the electrophysiological features ${X}^{ephys}\in {R}^{K*l}$ of $K$ samples and $l$ electrophysiological features. The model is trained to optimize the parameters based on the modified loss functions from Eqs. 8 and 9, and the updated loss function is shown in Eqs. 14 and 15.

14 $${\mathcal{L}}^{pri}\left(y, \widehat{y}\right)=\mathcal{L}\left( {f}_{\theta }^{pri}\left({X}^{GEX}, {X}^{ephys}\right), y\right)= -\frac{1}{K} {\sum }_{k=1}^{K}{y}_{k}\mathrm{log}({\widehat{y}}_{k})$$

15 $${\mathcal{L}}^{aux}\left({C}^{GEX}, {C}^{ephys}\right)= \frac{1}{K} {\sum }_{k=1}^{K}{\left({C}^{ephys}_{k}- {f}_{\theta }^{aux}\left({C}^{GEX}_{k}\right)\right)}^{2}$$

The overall objective loss function for the model is computed using Eq. 16.

16 $$\mathit{arg}\underset{\theta }{\mathrm{min}} ( {\mathcal{L}}^{pri}+ \lambda * {\mathcal{L}}^{aux})$$

## Results

### Classification of schizophrenia individuals from genotype and bulk gene expression data

We first evaluated the performance of DeepGAMI using population-level genotype and bulk-tissue gene expression data of schizophrenia individuals in the dorsolateral prefrontal cortex (DLPFC). We utilized PsychENCODE consortium [49] data for predicting schizophrenia (SCZ) versus healthy individuals. PsychENCODE contains 1866 individuals from several cohorts with different neuropsychiatric diseases. After filtering for schizophrenia and control samples with multimodal data, we ended up with 1168 samples from three cohorts: CommonMind (CMC, 565 samples), Lieber Institute for Brain Development (LIBD, 511 samples), and BrainGVEX (92 samples). We used the CMC cohort consisting of 343 control and 275 SCZ individuals for tuning and training the model. The CMC data was first split into train and held-out test sets with a ratio of 90:10. We then performed filtering, preprocessing, and feature selection (Methods and materials) on the training samples and ended up with 7433 SNPs, 208 transcription factors (TFs), and 2870 intermediate layer genes.

**Fig. 2:**
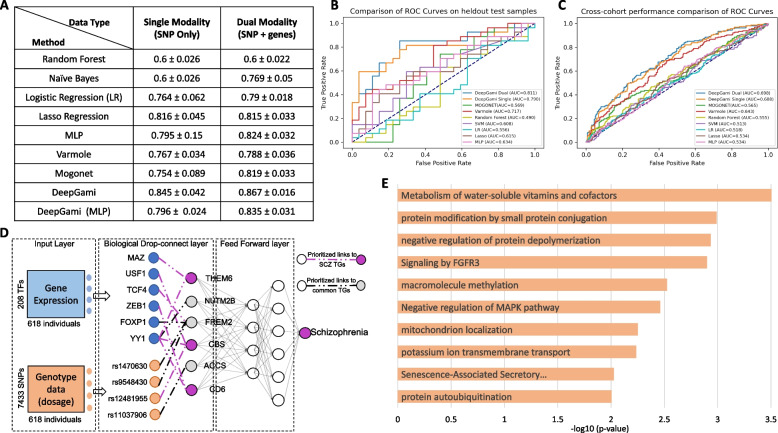
Schizophrenia classification and functional genomic prioritization using genotype and bulk-tissue gene expression data. The population data was from the PsychENCODE project (Methods and materials). **A** Balanced accuracies from 5-fold cross-validation and **B** receiver operating characteristic (ROC) curves of DeepGAMI dual-modality model (dark blue), DeepGAMI single modality model (orange), Lasso (brown), LR (light blue), Random Forest (yellow), SVM (purple), Multilayer perceptron (MLP, pink), Varmole (red), and MOGONET (green) for classifying schizophrenia vs. control individuals on the held-out test samples. **C** ROC curves of various methods on cross-cohort SCZ prediction. **D** Select examples of prioritized transcription factors, SNPs, target genes (latent features, and functional links (GRNs, eQTLs) for schizophrenia. Purple: known schizophrenia genes. **E** Function and pathway enrichments of prioritized schizophrenia SNPs

We performed fivefold cross-validation for selecting the optimal hyper parameters. Figure 2A shows the fivefold cross-validation balanced accuracies of DeepGAMI using both genotype and gene expression as inputs (Dual) and only genotype as input (Single) in comparison to other state-of-the-art classifiers along with DeepGAMI with no biological priors. DeepGAMI dual ($BACC=0.867\pm 0.016$) and DeepGAMI single ($BACC=0.845\pm 0.042$) achieved the highest performance in comparison with other methods. DeepGAMI with no biological prior ($BACC=0.835\pm 0.031$ for dual modalities and $BACC=0.796\pm 0.024$ for single modality) was the closest in performance.

After selecting the optimal hyperparameters through fivefold cross-validation, we built a model using these settings on the training samples and evaluated its performance on the held-out test samples within the same CMC cohort. DeepGAMI ($BAC{C}_{DUAL}=0.796$ and $BAC{C}_{SINGLE}=0.759$) outperformed other state-of-the-art methods ($BAC{C}_{Varmole}=0.7296$, $BAC{C}_{MOGONET}=0.593$, $BAC{C}_{LASSO}=0.537$, and $BAC{C}_{LR}=0.5556$, Additional file 1: Fig S1A). To further test the generalizability of DeepGAMI, we used combined SCZ samples from LIBD and BrainGVEX cohorts for independent validation. These cohorts contained 257 SCZ samples and 377 control samples. We trained our model on the CommonMind cohort and evaluated the performance on the LIBD and BrainGVEX samples. DeepGAMI was able to classify with $BAC{C}_{DUAL}=0.625$ and $BAC{C}_{SINGLE}=0.623$ outperforming other models: Varmole ($BACC=0.602$), Mogonet ($BACC=0.518$), MLP ($BACC=0.545$), LR ($BACC=0.519$), and Lasso ($BACC=0.547$) as shown in Additional file 1: Fig S1A. ROC curves of various models on held-out test samples and independent SCZ samples are shown in Fig. 2B and C respectively, where DeepGAMI has the best performance. Additionally, we evaluated the performance of DeepGAMI on 47 bipolar disorder (BPD) samples from the CMC cohort where we trained DeepGAMI on SCZ samples and tested on BPD samples. As we had only BPD samples, sensitivity measure was used as the performance metric. We found that DeepGAMI (${SENSITIVTY}_{DUAL}=0.6596$ and ${SENSITIVTY}_{SINGLE}=0.5957$) performed better than Varmole (${SENSITIVTY}_{Varmole}=0.4681$) and MOGONET (${SENSITIVTY}_{MOGONET}=0.4255$) as shown in Additional file 1: Fig S1B. This demonstrates the application of DeepGAMI for cross-disorder prediction.

We then used integrated gradient approach (Methods and materials) to prioritize SNPs, genes, and functional links on the held-out SCZ samples. Figure 2D shows a few examples of these DeepGAMI’s prioritized SNPs, genes, and links. Our model was able to prioritize SCZ-related genes like CBS [94, 95], THEM6 [96], and CD6 [97, 98] among others shown as pink circles in Fig. 2D. CBS (cystathionine beta-synthase) gene plays a significant role in reducing the level of homocysteine which is etiologically linked to SCZ. However, mutations in CBS leads to glia/astrocyte dysfunction which is associated with SCZ pathogenesis [94]. THEM6 gene has shown association with SCZ individual. Similarly, CD6 gene is related to immune system which in turn is associated with SCZ [97]. A complete list of prioritized SNPs and genes with the importance score is available in Additional files 3 and 4. We then used the prioritized functional links to extract the genes present in these links (213 genes, top-ranked 10% of the link importance scores). We performed enrichment analysis on these 213 genes with 3064 genes (total number of input features) as background using Metascape. We found several known functions and pathways related to SCZ, like signaling by FGFR3 [99, 100], negative regulation of MAPK pathway [101, 102], and senescence-associated secretory phenotype [103] (Fig. 2E).

### Clinical phenotype prediction and gene regulatory network prioritization in Alzheimer’s disease

To demonstrate the application of DeepGAMI for predicting complex clinical phenotypes, we ran DeepGAMI on Alzheimer’s disease (AD) cohort, ROSMAP [55], which contains multi-omics data from the brain DLPFC region. We used its bulk-tissue gene expression and genotype data for this analysis (Methods and materials). As the cohort contains data from the DLPFC brain region, we extracted the eQTL information from the GTEx consortium [88] from the same brain region (human brain frontal cortex, BA9), which contains 146,763 eQTL SNPs, and used the GRN from the PsychENCODE consortium [49]. Clinical phenotypes included in our analysis are cognitive diagnosis (COGDX), CERAD, and BRAAK scores (Methods and materials). Additional file 2: Table S3 summarizes the number of features and class labels for this analysis.

**Fig. 3:**
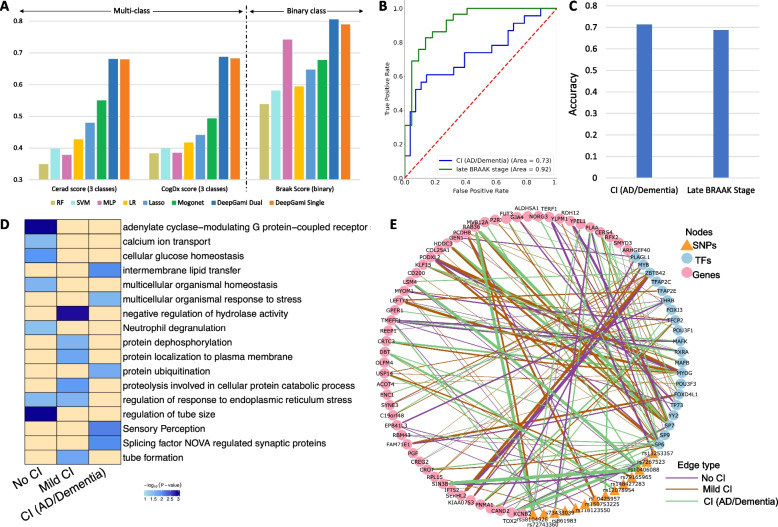
Multi-class clinical phenotype prediction and regulatory network prioritization in Alzheimer’s disease.** A** Fivefold cross-validation performance of DeepGAMI (Dual modality: dark blue, Single modality: orange) on three different phenotypes: neuritic plaque measure (CERAD score, multi-class), cognitive impairment (COGDX score, multi-class) and neurofibrillary tangle pathology (BRAAK stage, binary) in comparison with Lasso (brown), LR (light blue), Random Forest (yellow), SVM (purple), MLP (pink), and MOGONET (green). **B** ROC curves of held-out test samples for cognitive COGDX phenotype (blue) and late BRAAK stage (green). **C** Classification accuracies of the independent dataset for COGDX phenotype and BRAAK stage. **D** Enrichment analysis of prioritized genes for no cognitive impairment, mild cognitive impairment, and cognitive impairment (AD/Dementia) classes of COGDX phenotype.** E** Select an example of a prioritized regulatory network for the cognitive impairment phenotype. The edge thickness between any two nodes corresponds to the prioritized link importance score of the associated nodes. The edge color represents the three classes

We split the data into training and held-out test sets using an 80:20 ratio. We used a fivefold CV on the training set for tuning the hyperparameters and obtaining the best performance. DeepGAMI outperforms state-of-the-art classifiers ($BACC = 0.806$ for BRAAK, 0.689 for COGDX, and 0681 for CERAD, Fig. 3A, Additional file 2: Table S4). For BRAAK phenotype, DeepGAMI ($BACC=0.806 \pm 0.03$ for dual, $BACC=0.79 \pm 0.02$ for single) outperforms random guess (BACC = 0.50), Random Forest classifier($BACC=0.538 \pm 0.01$), SVM ($BACC=0.5808 \pm 0.13$), MLP ($BACC=0.742 \pm 0.02$), LR ($BACC=0.594 \pm 0.018$), Lasso ($BACC=0.647 \pm 0.057$), and MOGONET ($BACC=0.678 \pm 0.025$). Looking at a more complex phenotype with multi-class, DeepGAMI with two modalities improved the classification accuracy of COGDX ($BACC=0.688 \pm 0.07$) compared to the highest accuracy of the best model (MOGONET, $BACC=0.4938 \pm 0.05$). Also, DeepGAMI with single modality input improved the multi-class classification accuracy ($BACC=0.6826 \pm 0.07$) compared to MOGONET($BACC=0.4938 \pm 0.05$). Following this, we tested the performance of the classifiers with optimal hyperparameters on 100 randomly generated training and validation sets. Based on the k.s. test, both DeepGAMI dual and DeepGAMI single has the best performance (Additional file 1: Fig S2A and Fig S2B).

We tested DeepGAMI on additional samples from ROSMAP cohort with missing modalities where these individuals had only genotype information (single modality as input). These samples belonged to late-stage BRAAK individuals and CI (cognitive impairment in AD/Dementia). DeepGAMI was able to achieve AUC scores of 0.92 for late-stage BRAAK and 0.73 for CI (Fig. 3B). We were able to classify these late-stage BRAAK individuals with an accuracy of 0.687 and CI individuals with an accuracy of 0.713 (Fig. 3C). We observed that our model had higher AUC scores in comparison with accuracy scores for late-stage BRAAK samples. This might be due to the imbalance in the training samples. Also, AUC works best in the binary classification setting and has inconsistencies in the multi-class problem [104, 105].

We then performed enrichment analysis on the top-ranked genes that were regulated by the SNPs and TFs for each group (no CI, mild CI, CI) of the COGDX phenotype (Fig. 3D, Additional file 5) and generated a network containing the prioritized SNPs and TFs with prioritized links to the genes (Fig. 3E, Additional file 6). These prioritized genes were enriched with many known cognitive impairment functions and pathways. For example, controls were enriched for adenylate cyclase-modulating G protein-coupled receptors that are known to have a role in the pathological prognosis of AD [106, 107]. Mild CI was associated with protein dephosphorylation [108], response to endoplasmic reticulum stress [109–111] and proteolysis in cellular protein catabolic process [112]. We observed that CI was associated with sensory perception and splicing factor NOVA regulated synaptic proteins. Sensory perception impairment is known to affect cognition [113–115]. NOVA regulates genes critical for neuronal function [116] and known to affect patients with inhibitory motor control dysfunctions [117].

### Cortical layer classification for single-cell neuronal cells in mouse visual cortex

**Fig. 4:**
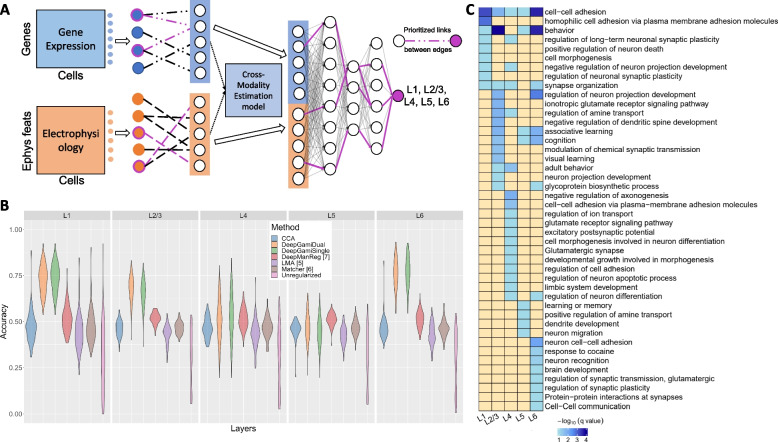
Classifying cellular phenotype in single neuronal cells of mouse visual cortex.** A** DeepGAMI model for cell layer classification.** B** Balanced accuracies for classifying cell layers in the mouse visual cortex by DeepGAMI dual-modality (orange), DeepgGAMI single-modality (green) versus DeepManReg [92] (dark pink), neural network classification without any regularization (light pink), LMA [118] (violet), CCA [119] (blue), and MATCHEr [120] (brown). **C** Gene enrichment analysis showing the enriched terms for layer-specific prioritized genes

We also tested DeepGAMI on additional non-omics modalities using an emerging Patch-seq dataset [81] containing single-cell multimodal data for the visual cortex brain region in neuronal cells of mouse species. This dataset includes transcriptomics and electrophysiological (ephys) data. We used the cell cortical layers (L1, L2/3, L4, L5, L6) that reveal the location of the cells in the visual cortex as the cellular phenotype. We followed the data extraction and preprocessing as done in DeepManReg [92] and ended up with 41 ephys features and 1000 genes for 3654 neuronal cells. Figure 4A depicts the overall architecture of DeepGAMI for this dataset. It demonstrates that DeepGAMI can handle multiple modalities besides genotype and gene expression and can also perform multi-class classification. While Fig. 2C and Fig. 4A looks similar, they serve different purposes, with Fig. 4A showing the model architecture and Fig. 2C highlighting the prioritization results.

To evaluate DeepGAMI’s performance on Patch-seq data, we adopted the validation technique used in previous studies [92] and randomly split the cells into training/testing sets with a ratio of 80:20 and obtained 100 different sets. As there was a huge imbalance in the number of cells for each layer (L1: 262 cells; L2/3: 1097 cells; L4: 385 cells; L5: 1176 cells; L6: 734 cells), we applied SMOTE [121] oversampling technique on the training set to have a balanced number of cells for each layer in the training label and ended up with 941 cells in each layer. SMOTE identifies k-nearest neighbors of each sample in the minority class and creates synthetic samples along the line segments joining these neighbors. Biological Dropconnect was not used as there was no prior biological data available. Thus, we instead used full connectivity, where each gene and ephys feature had an association with the intermediate latent space layer. After various parameter tuning, the latent space dimension was set to 500.

We compared the prediction accuracy of DeepGAMI with different methods using pairwise Kolmogorov–Smirnov test (k.s test) on the accuracy distribution over 100 runs. DeepGAMI has higher prediction accuracy for classifying cell cortical layers than other methods (k.s test statistic = 1, $p-value<2.2{e}^{-16}$ for dual-modality input, k.s test statistic = 1, $p-value<1.9{e}^{-16}$ for single modality gene expression input, Fig. 4B and Additional file 2: Table S5). Furthermore, the average accuracy of DeepGAMI dual-modality mode (0.6571 with a 95% confidence interval (CI) of [0.6249, 0.6892]) and DeepGAMI single modality mode (0.6463 [0.6129, 0.6797]) is higher than the random guess baseline of 0.2 (five labels), LMA [118] (0.43 [0.322, 0.496]), CCA [119] (0.462, [0.401, 0.513]), MATCHER [120] (0.465, [0.409, 0.528]), and DeepManReg [92] (0.514, [0.479, 0.548])).

We then compared the performance of DeepGAMI with- and without- oversampling on multi-class classification. We hypothesized that oversampling helps perform better on imbalanced dataset. DeepGAMI with oversampling had higher accuracies for classes L1, L4, and L6 while if performed slightly lesser on layers L2/3 and L5 (Additional file 1: Fig S3A). DeepGAMI with oversampling has two times better accuracy for cell layer L4 which had the least number of samples. We also converted the multi-class labels into binary classification problem. We then developed DeepGAMI for each cell layer without oversampling. As expected, DeepGAMI had higher accuracy on binary classification problem (Additional file 1: Fig S3B). While binary classification has higher accuracies, multiclass classification is useful when classifying new cells into the cell layers.

We extracted 112 (L4) neuronal cells patch-seq data [122] containing gene expression data but only a small set of electrophysiological data for independent testing. We applied DeepGAMI for classifying these 112 cells by giving only gene expression (single modality mode) and allowing the model to estimate the latent space of ephys features. For the negative samples required for performance estimation, we used the predictions on the motor cortex data [81]. The motor cortex dataset contains 1286 genes and 29 electrophysiological features of neuronal cells without the L4 layer: L1, L2/3, L5, and L6. After predicting these cells, we used the predicted values for the L4 layer as the negative samples, combined them with the predictions for the L4 layer for the 112 samples, and computed the AUC score. DeepGAMI classified the cells into L4 layer with an AUC score of 0.73.

Following the prediction, we extracted the top 10% of the prioritized genes and importance scores of the 41 ephys features for each cell layer (Additional file 7). Figure 4C shows the gene set enriched terms [86] for each layer. Enriched terms like cell–cell adhesion and neuron projection development appear in all layers [123]. Layer 4 is enriched with excitatory neurons and their activities [93, 124]. Many groups were enriched for behavior (especially L2 and L6), and synapse organization. L1 and L2/3 groups were enriched for negative regulation of neuron projection development and long-term neuronal synaptic plasticity regulation. The upper layers of the cortex (groups L2/3 and L4) were enriched for amine transport regulation and adult behavior; the primary input from the thalamus goes to Layer 4, whose input then goes to Layers 2 and 3. Additional file 1: Fig S4 compares the importance scores of the 41 ephys features across six cell layers.

### Classification of schizophrenia individuals using genotype and cell-type gene expression data

**Fig. 5:**
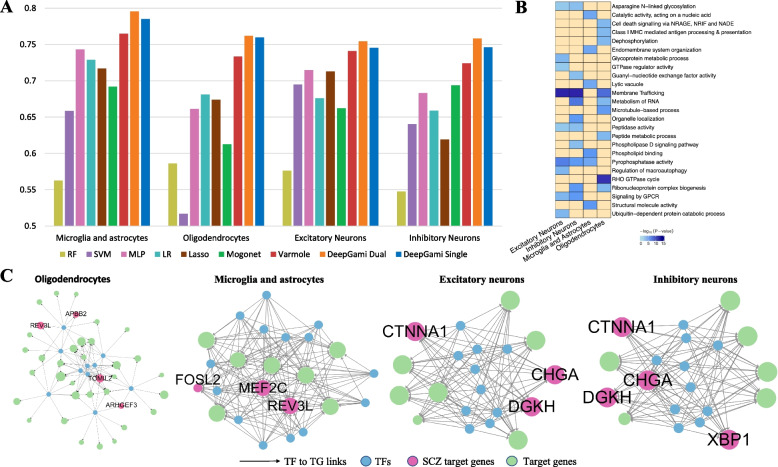
Classification of schizophrenia individuals and prioritization of genes, SNPs, and regulatory network using genotype and gene expressions of four cell types (microglia and astrocytes, oligodendrocytes, inhibitory neurons, and excitatory neurons).** A** Balanced accuracies from 5-fold cross-validation of DeepGAMI dual-modality model (dark blue), DeepGAMI single modality model (orange) in comparison with Lasso (brown), LR (light blue), Random Forest (yellow), SVM (purple), MLP (pink), Varmole (red), and MOGONET (green). **B** Pathway enrichment of prioritized schizophrenia SNPs for four cell types. The blue shade gives the − log(*p*-values) of the term. **C** Prioritized cell-type gene regulatory networks with pink circles representing schizophrenia genes. The size of the target gene is defined by the number of prioritized links between the SNPs and the associated gene

We also tested if DeepGAMI can prioritize cell-type disease genes and SNPs in major brain cell types for schizophrenia: excitatory neurons, inhibitory neurons, oligodendrocytes, and other glia (microglia and astrocytes). Notably, we used genotype and cell-type-specific gene expression imputed using bMIND [89] from a reference panel of four cell types: GABAergic (i.e., inhibitory) neurons, glutamatergic (i.e., excitatory) neurons, oligodendrocytes, and a remaining group composed mainly of microglia and astrocytes. Additional file 2: Table S6 summarizes the number of features used as input for the model for these four cell types. DeepGAMI classified SCZ individuals with a balanced accuracy of $0.795\pm 0.035$ for dual-modality and $0.784\pm 0.024$ for single modality for microglia and astrocyte cell type in comparison to $0.563\pm 0.049$ for RF, $0.6585\pm 0.079$ for SVM, $0.7429\pm 0.058$ for MLP, $0.729\pm 0.033$ for LR, $0.717\pm 0.031$ for Lasso, $0.765 \pm 0.026$ for Varmole, and $0.6919\pm 0.037$ for MOGONET. Similarly, DeepGAMI performs better fivefold classification than other models for the other three cell types (Fig. 5A, Additional file 2: Table S7). Additional file 1: Fig S5A compares the robustness of DeepGAMI with other state-of-the-art methods. DeepGAMI consistently outperformed Varmole, Mogonet, and other machine learning methods in all cell types. DeepGAMI was also able to produce a better classification of schizophrenia samples against control on held-out test data in comparison to existing methods (Additional file 1: Fig S5B).

We then performed enrichment analysis for the prioritized SNPs for each cell type. We found various known cell-type pathways and functions associated with SCZ (Fig. 5B) like organelle localization in inhibitory neurons [125], structural molecule activity in microglia and astrocytes [126], RHO GTPase cycle in oligodendrocytes [127], and regulation of macroautophagy in excitatory neurons [128]. We also found common SCZ-associated functional pathways across cell types like asparagine N-linked glycosylation [129, 130], membrane trafficking [131], and signaling by GPCR [132].

Figure 5C visualizes subnetworks of prioritized cell-type regulatory networks showcasing the ability of DeepGAMI to prioritize cell-type genes like APBB2 and TOMIL2 for oligodendrocytes, FOSL2 and MEF22C for microglia and astrocytes, and XBP1 for inhibitory neurons, and some common genes like REV3L (oligodendrocytes and microglia and astrocytes) and CTNNA1 (excitatory and inhibitory neurons). A complete list of importance scores of genes and SNPs for each cell type is available in Additional files 8 and 9.

### Assessing the impact of different networks as input on the classification performance

One of the major contributions of this study is the use of prior biological knowledge to guide the neural network model for phenotype prediction. To test the effectiveness of these prior networks (GRNs and eQTLs), we performed an ablation study of DeepGAMI, where we compared our proposed model with two additional variants: (1) DeepGAMI Random—We generated random networks in place of prior biological networks and used these as the basis for DropConnect and (2) DeepGAMI Bernoulli—Based on the number of edges in the GRNs and eQTLs, we used Bernoulli distribution to generate networks with the same distribution as the original networks where existing edges has an 80% chance of being selected where other links have 20% chance of being selected. We compared the performance of these three variations on the bulk tissue SCZ cohort, inhibitory neuron cell type SCZ cohort, and AD cohort with CERAD score phenotype. We performed 5-fold cross-validation to assess the performance of all the variations. As shown in Additional file 1: Fig S6, DeepGAMI, with prior biological knowledge, outperformed its variations in all classification tasks. DeepGAMI Bernoulli variations had a better performance than DeepGAMI random, indicating that a biology-guided neural network helps improve phenotype prediction and aids in prioritizing molecular and cellular features.

## Discussion

DeepGAMI is a novel interpretable deep learning model for improving genotype–phenotype prediction from multimodal data. Its auxiliary learning layer enables cross-modal imputation to predict phenotypes still when some modalities are unavailable. The model also takes prior biological information for aiding in prioritizing multimodal features (e.g., SNPs, genes) and feature networks (e.g., gene regulatory networks) related to the phenotypes.

As brain phenotypes involve complex cellular and molecular mechanisms, genotype and gene expression are a few of the many factors associated with mechanisms. We have demonstrated that DeepGAMI can handle various multimodal data as input in two scenarios. In the first scenario, DeepGAMI was able to accurately predict cortical layers using gene expression and electrophysiological features in mouse visual cortex (Fig. 4). In the second scenario, we used DNA methylation and gene expression as inputs to DeepGAMI to predict AD phenotype (COGDX scores: No CI vs Mild CI vs CI-AD/Dementia) from the ROSMAP cohort. After preprocessing and filtering, we used 1198 CpG sites as features from the methylation data and 183 TF gene expressions as inputs. The intermediate gene layers consist of 1013 target genes. We used CpG island sites for each gene and GRN as biological priors. Notably, DeepGAMI achieved the best multi-class accuracy of 0.524 on the held-out test samples and 0.557 on the independent samples that only had methylation data (Additional file 1: Fig S7). Integrating additional modalities into DeepGAMI enables a more profound understanding of such mechanisms. For example, several studies have tried integrating copy number variations with DNA methylation [133], gene expression [134], and clinical data [135]. Trevino et al. [136] integrated RNA-seq and ATAC-seq over a period of time, studying various genetic activities and disease susceptibility in various neuropsychiatric disorders. MVIB [137] integrated gut microbial markers and abundance scores to classify various diseases. Furthermore, DeepGAMI currently integrates two data modalities. We plan to extend DeepGAMI to integrate more than two modalities in the future.

DeepGAMI uses fivefold cross-validation balanced accuracies and AUC scores to compare the performance of the model on various datasets. While cross-validation can be misleading when used for model selection, it can still be a useful technique for estimating the expected performance of the model on the test set and comparing results when the sample size is not large enough to split the data into separate train and test sets [138, 139]. Moreover, despite the relatively lower sample sizes, DeepGAMI has demonstrated accurate performance on held-out test samples for three cohort datasets: SCZ (Fig. 2B, C), AD (Fig. 3B, C), and mouse visual cortex (Fig. 4C) that only contain genotype information.

We evaluated the scalability of DeepGAMI by varying the number of input features to the model and recording the runtime. For this analysis, we used PsychENCODE consortium [49] data for predicting schizophrenia (SCZ) versus healthy individuals. Additional file 1: Fig S8 gives a detailed comparison of three models: DeepGAMI, Varmole, and MOGONET. We varied the number of input features from 100 to 100,000 and recorded the total runtime. We see that the runtime (training time) scales as the number of features increases. While the runtime performance is similar among all three models, MOGONET has the least runtime. We believe the cross-modal imputation layer of DeepGAMI might be the cause for relatively slower runtime.

One of the major contributions of DeepGAMI is its ability to make accurate phenotype predictions even when some modalities are missing, which is achieved using cross-modal imputation. DeepGAMI differs from traditional cross-modal imputation methods. Firstly, it aims to integrate multimodal data for improving phenotype prediction, rather than focusing solely on cross-modal imputation. Secondly, it can handle both lower sample size population-level datasets and single-cell multimodal datasets while the latter methods are mainly based on single-cell datasets. Lastly, existing cross-modal imputation techniques can be extended to perform various supervised and unsupervised learning tasks. However, it is important to note that this process involves building two separate models: one for modality estimation and another for prediction. As a result, there is a risk of missing phenotype-related shared features between modalities, which can potentially impact the accuracy of the predictions. Furthermore, traditional cross-modal imputation methods often do not allow for the incorporation of prior biological knowledge into the models. This can limit the interpretability of the results. DeepGAMI uses linear regression for cross-modal imputation. Even though our applications have shown the imputations work well, DeepGAMI can use nonlinear functions for cross-modality imputation, aiming to have nonlinear auxiliary learning. It is also possible to integrate existing cross-modal imputation methods into DeepGAMI’s auxiliary task of cross-modality imputation. This could potentially lead to further improvements in performance. The results from our analysis on AMP-AD data may be susceptible to batch effects and data-source-specific effects as we were able to only extract the ROSMAP cohort. Additional cohorts from the same study can further enhance the generalizability of our model.

Disease variation is affected by both genetic and non-genetic factors which can include covariates like age, batch, sex, and ethnicity. In many cases, the covariate information is either missing or partially available. The genotype data can be adjusted by these covariates by the users. We test the effect of three covariates (age, gender, and ethnicity) on the CMC cohort. For each SNP, we regress out its genotype data across individuals by covariates and then input the residuals to DeepGAMI and the classification performance decreased ($AU{C}_{dual} = 0.748$ and $AU{C}_{single} = 0.723$). DeepGAMI has higher performance with original genotype data, suggesting that the nodes in the hidden layers of DeepGAMI can capture the hidden effects of these covariates.

We showcase the ability of DeepGAMI in predicting phenotypes with genotype data alone. PRS is a popular regression-based method to quantify genotype to phenotype association. Thus, we calculated PRS for SCZ (binary trait) using three different methods: PLINK [140], LDpred2 [141], and PRSice [142] on our data. We used 7433 SNPs along with age, gender, and ethnicity as covariates for this analysis. PRS was able to explain moderate percentages of variations (${R}_{PLINK}^{2}$ = 0.584, ${R}_{LDpred2}^{2}$ = 0.567, and ${R}_{PRSice2}^{2}$ = 0.762). As AUC is typically used for evaluating classification problems, out result show that DeepGAMI dual ($AUC=0.895$) and DeepGAMI single ($AUC=0.867$) perform better in comparison to the recently reported PRS score for SCZ ($AUC=0.61$) [76] and heritability of SCZ (0.8) [143]. In future, DeepGAMI can be extended to integrate regression and predict continuous phonotypes like PRS.

## Conclusions

In this study, we presented DeepGAMI, an interpretable biology-guided deep learning framework for phenotype prediction using multi-modal data. We demonstrated that DeepGAMI improves prediction of disease types and clinical phenotypes and prioritizes phenotypic genomic features and regulatory networks in AD and SCZ, especially at the cell-type level. We envision DeepGAMI can be used to decipher functional genomics and gene regulation for other complex diseases.

### Supplementary Information

**Additional file 1: Fig S1.** Independent validation performance comparison on schizophrenia cohort with genotype and bulk tissue gene expression.** Fig S2.** Kolmogorov-smirnov (k.s.) test comparison of classification accuracy for Alzheimer’s disease cohort. **Fig S3.** Performance comparison of DeepGAMI with oversampling, without- oversampling, and binary classification on Patch-seq mouse visual cortex data. **Fig S4.** Integrated Gradient results for Patch-seq mouse visual cortex data.** Fig S5.** Independent validation performance comparison on schizophrenia cohort with genotype and celltype gene expression.** Fig S6.** Performance of DeepGAMI with its variations on ablation study across all classification tasks.** Fig S7.** Multiclass classification of AD phenotype (COGDX score: No CI, Mild CI, and CI) using methylation and gene expression data from ROSMAP cohort. **Fig S8.** Runtime comparison of DeepGAMI with MOGONET and Varmole on varying input feature sizes.

**Additional file 2: Table S1.** List of all hyperparameters used in DeepGAMI. **Table S2.** Summary table of the total number of trainable parameters for each dataset. **Table S3.** Summary table showing features and class labels for different available phenotypes for ROSMAP AD dataset. **Table S4.** Balanced accuracy comparison for ROSMAP AD dataset. **Table S5.** Balanced accuracy comparison for Patch-seq dataset. **Table S6.** Summary table of the features for cell-type-specific Schizophrenia dataset. **Table S7.** Binary Classification results for cell-type-specific Schizophrenia dataset.

**Additional file 3.** Prioritized bulk genes and SNPs for schizophrenia.

**Additional file 4.** Prioritized bulk transcription factors to genes and SNPs to gene links for schizophrenia.

**Additional file 5.** Prioritized genes and SNPs for cognitive impairment phenotype in Alzheimer’s disease.

**Additional file 6.** Prioritized transcription factors to genes and SNPs to gene links for cognitive impairment phenotype in Alzheimer’s disease.

**Additional file 7.** Prioritized genes and electrophysiological features for cell cortical layers in mouse visual cortex.

**Additional file 8.** Prioritized cell-type genes and SNPs for schizophrenia.

**Additional file 9.** Prioritized cell-type transcription factors to genes and SNPs to gene links for schizophrenia.

## Data Availability

The PsychENCODE bulk gene expression file for schizophrenia disorder can be downloaded from http://resource.psychencode.org/Datasets/Derived/DER-01_PEC_Gene_expression_matrix_normalized.txt [49], and the genotype data can be accessed from http://resource.psychencode.org [49]. The cell-type-specific reference panel used for gene expression imputation is available at https://www.synapse.org/#!Synapse:syn22321061 [144], and the imputed gene expression is available at https://www.synapse.org/#!Synapse:syn23234712 [89, 145]. ROSMAP Alzheimer’s disease gene expression data is available at https://doi.org/10.7303/syn3388564 [55] and genotype can be downloaded from https://doi.org/10.7303/syn3157329 [55]. The processed gene expression and electrophysiological data from Patch-seq in mouse visual cortex is available at https://github.com/daifengwanglab/scMNC [146]. DeepGAMI was implemented in Python using PyTorch as the deep learning package and the source code is publicly available at https://github.com/daifengwanglab/DeepGAMI [147].

## Abbreviations

GRN: Gene regulatory network
eQTL: Expression quantitative trait loci
GWAS: Genome-wide association studies
SCZ: Schizophrenia
AD: Alzheimer’s disease
CI: Cognitive impairment
MLP: Multilayer perceptron
PRS: Polygenic Risk Score
SNP: Single-nucleotide polymorphism
