# Supplementary material for: DeepGAMI: deep biologically guided auxiliary learning for multimodal integration and imputation to improve genotype–phenotype prediction
Source: Genome Med. 2023 Oct 31;15:88. doi: 10.1186/s13073-023-01248-6 (PMC10617196; doi:10.1186/s13073-023-01248-6)
Supplement: Supplementary file 1 — Additional file 1: Fig S1. Independent validation performance comparison on schizophrenia cohort with genotype and bulk tissue gene expression. Fig S2. Kolmogorov-smirnov (k.s.) test comparison of classification accuracy for Alzheimer’s disease cohort. Fig S3. Performance comparison of DeepGAMI with oversampling, without- oversampling, and binary classification on Patch-seq mouse visual cortex data. Fig S4. Integrated Gradient results for Patch-seq mouse visual cortex data. Fig S5. Independent validation performance comparison on schizophrenia cohort with genotype and celltype gene expression. Fig S6. Performance of DeepGAMI with its variations on ablation study across all classification tasks. Fig S7. Multiclass classification of AD phenotype (COGDX score: No CI, Mild CI, and CI) using methylation and gene expression data from ROSMAP cohort. Fig S8. Runtime comparison of DeepGAMI with MOGONET and Varmole on varying input feature sizes. [file 13073_2023_1248_MOESM1_ESM.docx]

**
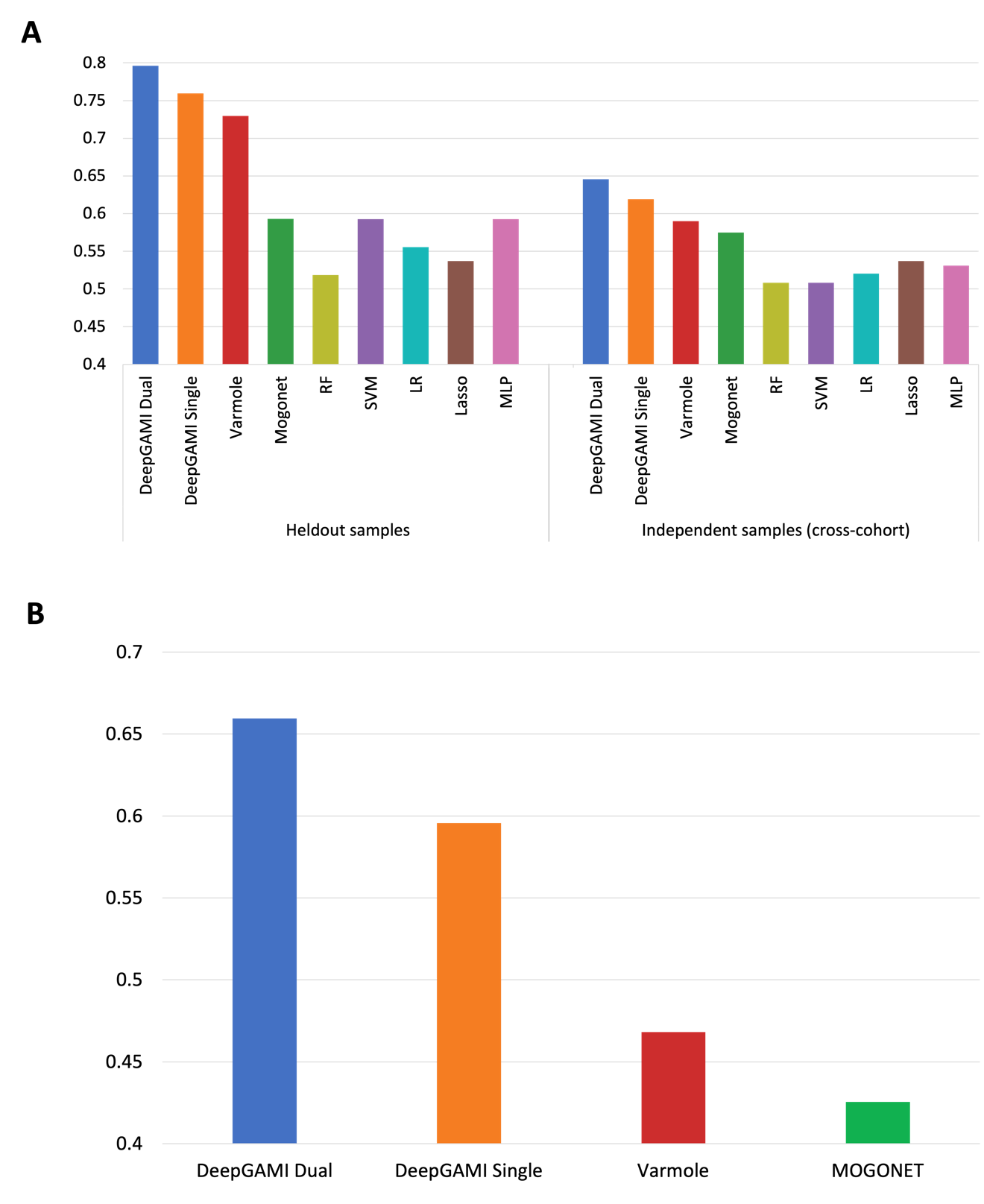
**

**Fig S1 – Independent validation performance comparison on schizophrenia cohort with genotype and bulk tissue gene expression. (A)** Balanced accuracy scores comparison of various models on held-out samples (54 within-cohort samples) and independent cross-cohort samples (511 LIBD + 92 BrainGVEX) **(B)** Comparison of sensitivity scores of various models on 47 bipolar samples. Training was performed on SCZ samples and testing was done on bipolar disorder samples.

**
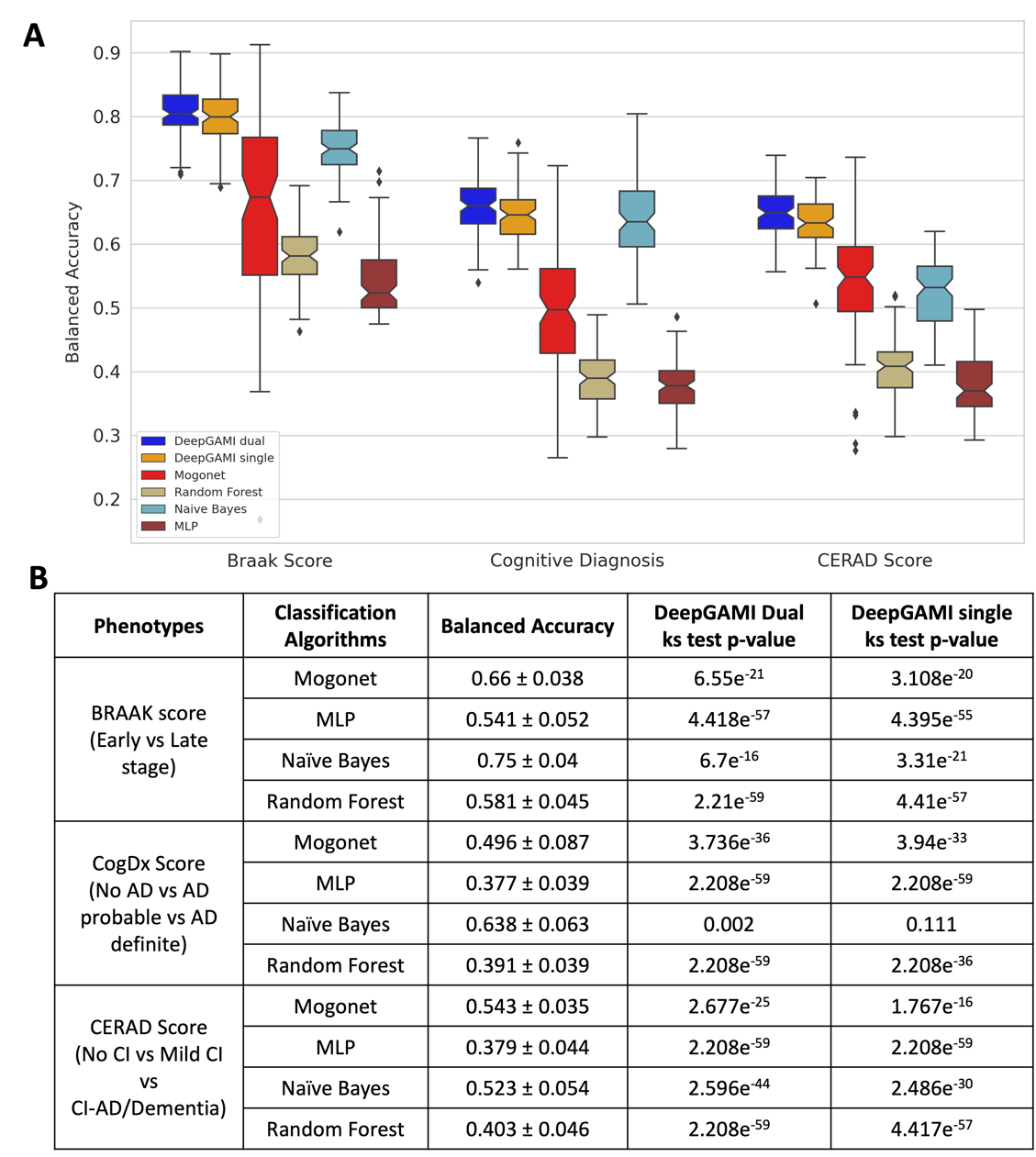
Fig S2 – Kolmogorov-smirnov (k.s.) test comparison of classification accuracy for Alzheimer’s disease cohort. (A**) Comparison of classification performance on 100 randomly generated train and validation sets. **(B)** k.s. test statistics comparison of DeepGAMI dual and DeepGAMI single with other existing approaches.

**
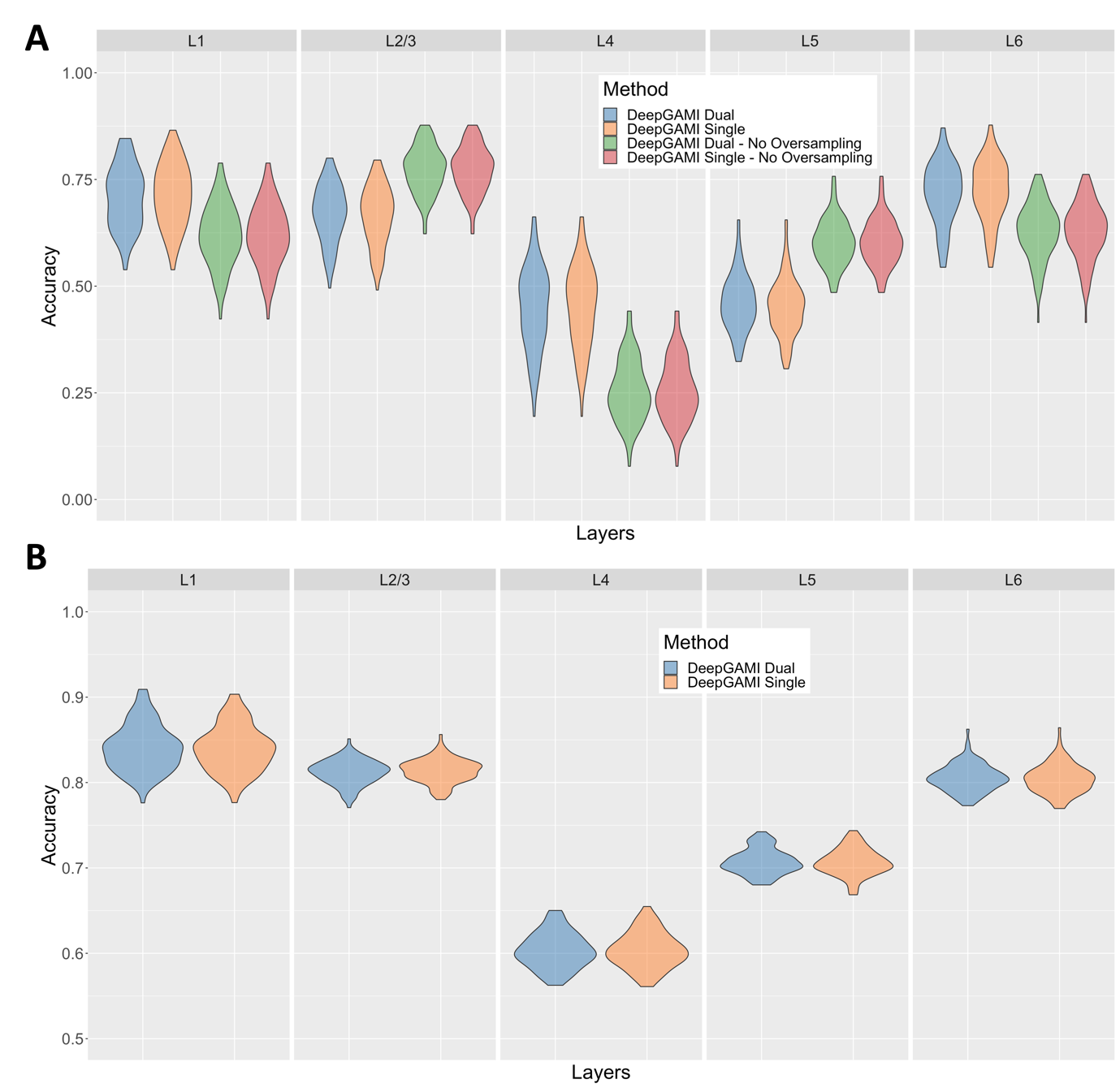
**

**Fig S3 – Performance comparison of DeepGAMI with oversampling, without- oversampling, and binary classification on Patch-seq mouse visual cortex data. (A)** Balanced accuracies for classifying cell layers in the mouse visual cortex by DeepGAMI dual-modality (blue), DeepgGAMI single-modality (orange) versus DeepGAMI dual-modality without-oversampling (green) and DeepGAMI single-modality without-oversampling (pink).  **(B)** Binary classification accuracies of DeepGAMI dual-modality (blue) and single-modality(orange) on five cell layers using one versus all approach.


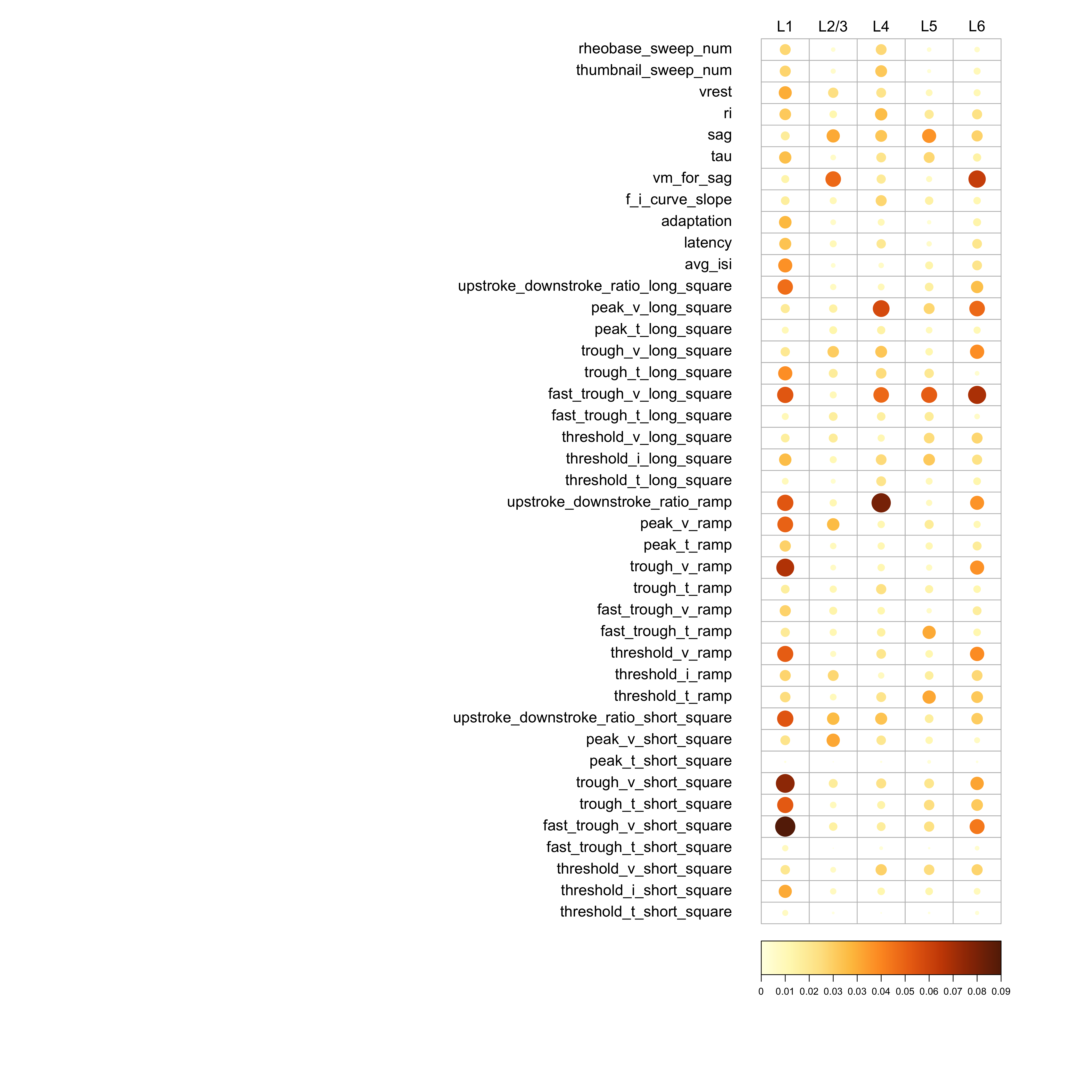

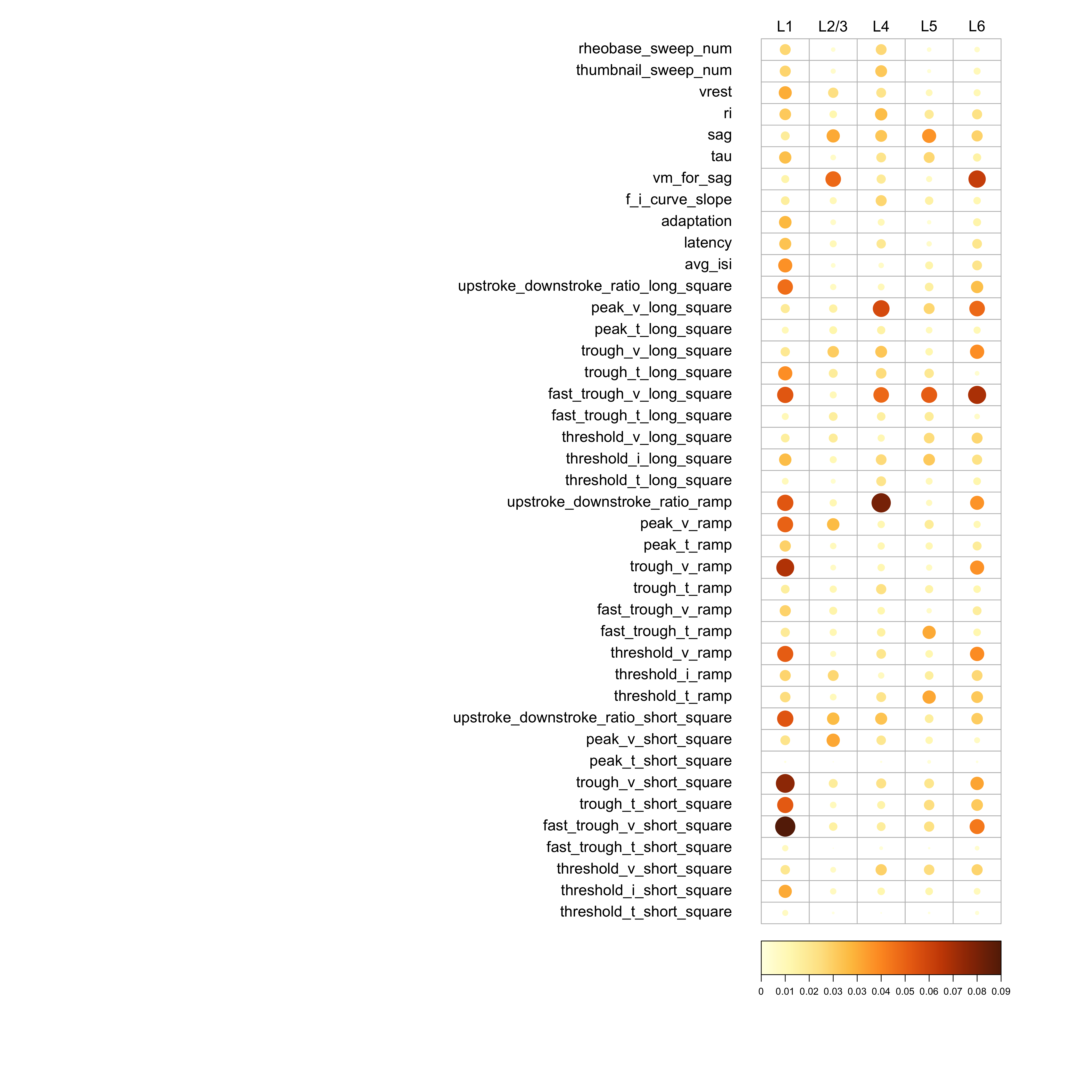


**Fig S4 – Integrated Gradient results for Patch-seq mouse visual cortex data.** Corrplot comparing the importance score of all 41 electrophysiological features across the five cell layers derived from DeepGAMI.

Importance Score

**
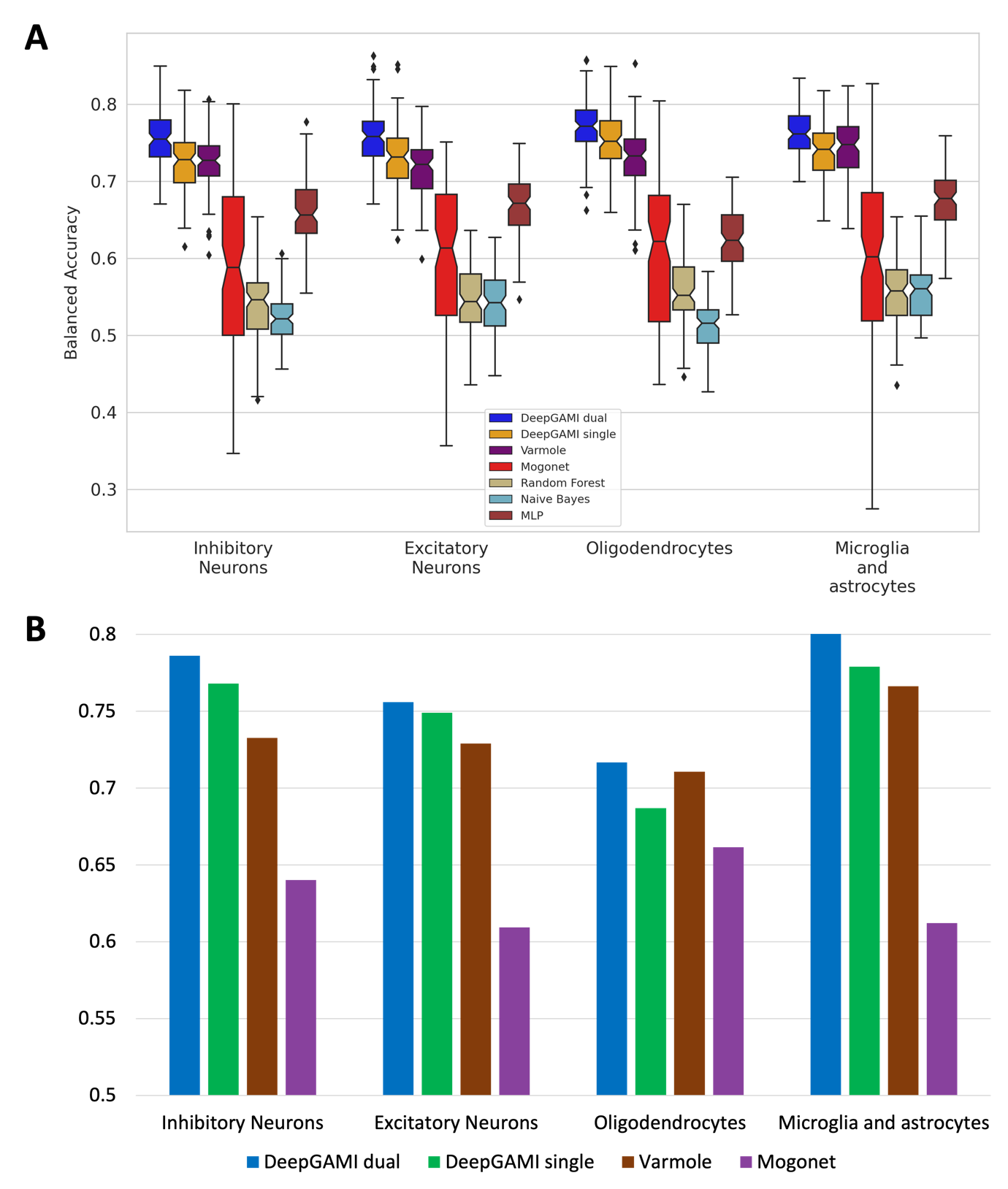
**

**Fig S5 – Independent validation performance comparison on schizophrenia cohort with genotype and celltype gene expression. (A)** Comparison of classification performance on 100 randomly generated test and validation set. **(B)** Balanced accuracy scores of various models on held-out samples.

**
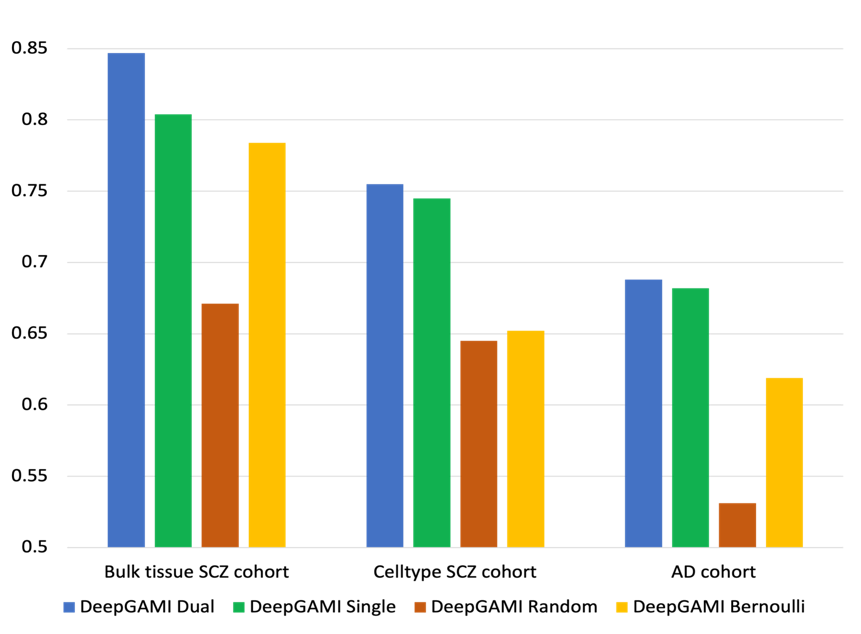
**

**Fig S6 – Performance of DeepGAMI with its variations on ablation study across all classification tasks.**

**
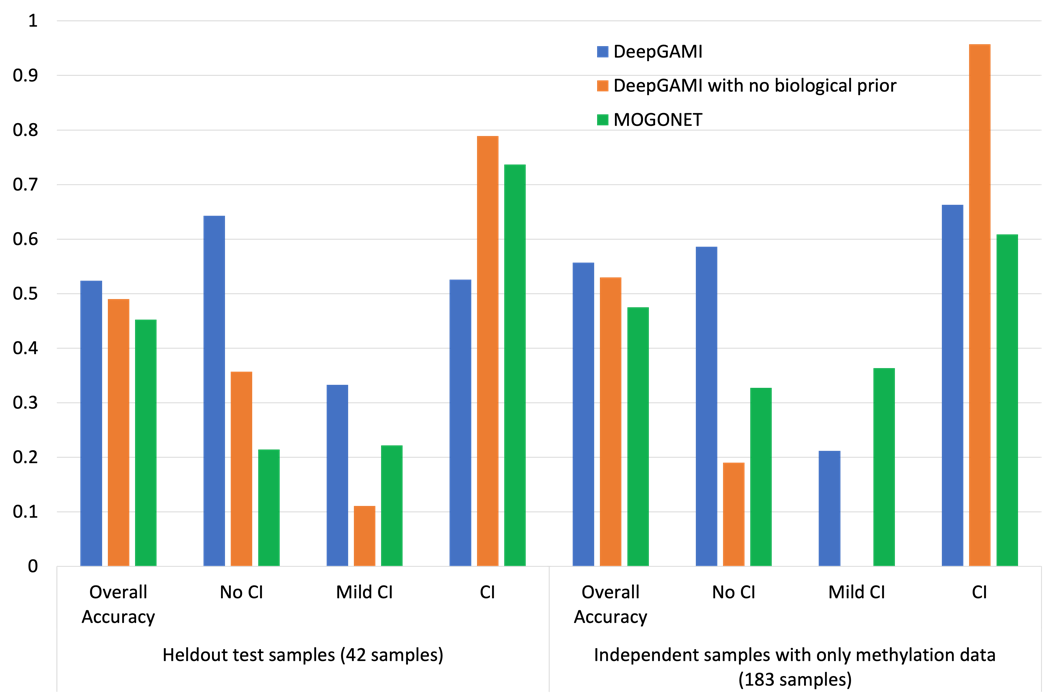
**

**Fig S7 – Multiclass classification of AD phenotype (COGDX score: No CI, Mild CI, and CI) using methylation and gene expression data from ROSMAP cohort.**  The figure shows overall accuracy comparison as well as individual class comparison of various models. For this analysis, we used 1198 CpG sites as features for the methylation data and 183 gene expressions of TFs. The intermediate gene layers consist of 1013 target genes. We used CpG island sites for each gene and GRN as biological priors.

**
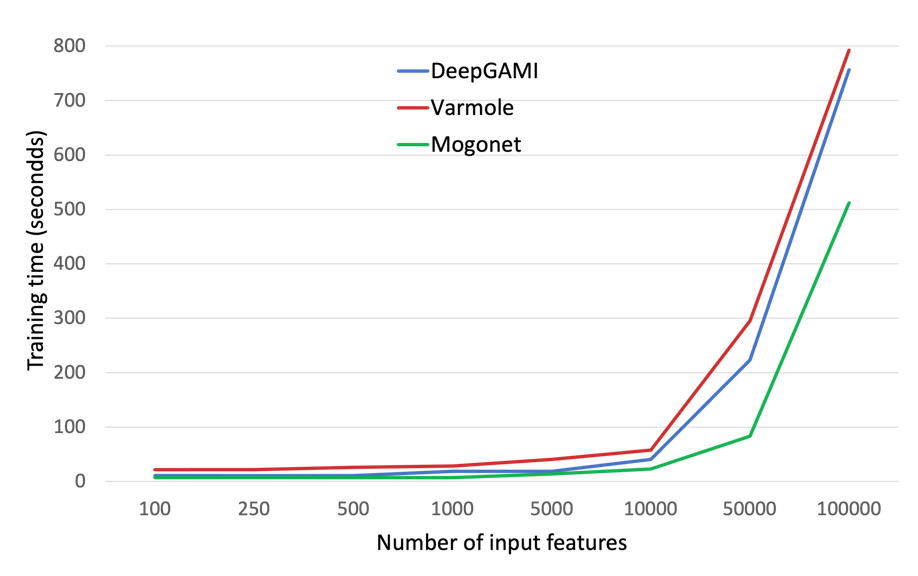
**

**Fig S8 – Runtime comparison of DeepGAMI with MOGONET and Varmole on varying input feature sizes.**
