# Supplementary material for: DeepGAMI: deep biologically guided auxiliary learning for multimodal integration and imputation to improve genotype–phenotype prediction
Source: Genome Med. 2023 Oct 31;15:88. doi: 10.1186/s13073-023-01248-6 (PMC10617196; doi:10.1186/s13073-023-01248-6)
Supplement: Supplementary file 2 — Additional file 2: Table S1. List of all hyperparameters used in DeepGAMI. Table S2. Summary table of the total number of trainable parameters for each dataset. Table S3. Summary table showing features and class labels for different available phenotypes for ROSMAP AD dataset. Table S4. Balanced accuracy comparison for ROSMAP AD dataset. Table S5. Balanced accuracy comparison for Patch-seq dataset. Table S6. Summary table of the features for cell-type-specific Schizophrenia dataset. Table S7. Binary Classification results for cell-type-specific Schizophrenia dataset. [file 13073_2023_1248_MOESM2_ESM.docx]

| **Hyper-parameters** | **Values** |
| --- | --- |
| Number of latent dimensions | {250, 500, 1000} |
| Number of hidden layers | {1, 2, 3} |
| Number of neurons | {50, 100, 250, 500, 1000} |
| Dropout rate | {0.25, 0.5, 0.75} |
| L2 regularization rate | {0.0001, 0.001, 0.01, 0.1} |
| Learning rate | {0.0001, 0.001, 0.01, 0.1} |
| λ (Auxiliary loss regularization) | {0.25, 0.5, 1} |

**Table S1 - List of all hyperparameters used in DeepGAMI**

**Table S2 – Summary table of the total number of trainable parameters for each dataset.**

| Cohort | Phenotype | Celltype | Number of trainable parameters |
| --- | --- | --- | --- |
| Schizophrenia | SCZ vs Control | Bulk | 1204203 |
|  | SCZ vs Control | Inhibitory neurons | 77027 |
|  | SCZ vs Control | Excitatory neurons | 59625 |
|  | SCZ vs Control | Microglia and Astrocytes | 106583 |
|  | SCZ vs Control | Oligodendrocytes | 52211 |
| Alzheimer’s | Braak Score | Bulk | 278263 |
|  | Cerad Score | Bulk | 580228 |
|  | CogDx score | Bulk | 452413 |
| Mouse Visual Cortex | Cell layer | Neuronal cell (single cell) | 114607 |

**Table S3 - Summary table showing features and class labels for different available phenotypes for ROSMAP AD dataset.**

| **Phenotype** | **TFs** | **SNPs** | **Genes** | **Class Labels** |
| --- | --- | --- | --- | --- |
| COGDX score | 102 | 273 | 354 | No CI, Mild CI, and CI (AD/dementia) |
| CERAD score | 98 | 467 | 369 | No AD, AD probable, and AD definite |
| BRAAK staging | 114 | 544 | 366 | Early stage and late stage |

**Table S4 – Balanced accuracy comparison for ROSMAP AD dataset.**  The table compares performance of DeepGAMI with other baseline ML methods for three different phenotypes: CERAD score, COGDX score, and BRAAK staging

|  | **CERAD score (3 classes)** | **COGDX score (3 classes)** | **BRAAK Staging (binary)** |
| --- | --- | --- | --- |
| Random Forest | 0.35 | 0.383 | 0.538 |
| Naïve Bayes | 0.445 | 0.34 | 0.555 |
| MLP | 0.378 | 0.385 | 0.742 |
| DeepGami Dual | 0.681 | 0.688 | 0.806 |
| DeepGami Single | 0.68 | 0.682 | 0.79 |

**Table S5 – Balanced accuracy comparison for Patch-seq dataset.**  The table compares five-fold cross-validation balanced accuracies of DeepGAMI with other several ML methods across five cell layers for visual cortex region in mouse brain.

|  | **L1** | **L2/3** | **L4** | **L5** | **L6** |
| --- | --- | --- | --- | --- | --- |
| Unregularized | 0.308 ± 0.177 | 0.312 ± 0.155 | 0.314 ± 0.169 | 0.302 ± 0.157 | 0.3 ± 0.155 |
| DeepManReg | 0.515 ± 0.071 | 0.518 ± 0.031 | 0.514 ± 0.055 | 0.511 ± 0.034 | 0.511 ± 0.047 |
| LMA | 0.439 ± 0.093 | 0.431 ± 0.051 | 0.435 ± 0.073 | 0.425 ± 0.05 | 0.428 ± 0.059 |
| CCA | 0.467 ± 0.079 | 0.462 ± 0.042 | 0.466 ± 0.053 | 0.459 ± 0.041 | 0.462 ± 0.056 |
| Matcher | 0.471 ± 0.086 | 0.466 ± 0.032 | 0.466 ± 0.055 | 0.465 ± 0.041 | 0.463 ± 0.046 |
| DeepGami Dual | 0.75 ± 0.076 | 0.689 ± 0.067 | 0.564 ± 0.095 | 0.484 ± 0.085 | 0.797 ± 0.075 |
| DeepGami Single | 0.765 ± 0.073 | 0.648 ± 0.069 | 0.61 ± 0.094 | 0.443 ± 0.083 | 0.815 ± 0.068 |

**Table S6 – Summary table of the features for cell-type-specific Schizophrenia dataset**

| **Cell type** | **TFs** | **SNPs** | **Genes** |
| --- | --- | --- | --- |
| Oligodendrocytes | 247 | 339 | 66 |
| Microglia | 552 | 231 | 108 |
| Inhibitory neurons | 465 | 206 | 88 |
| Excitatory neurons | 414 | 198 | 73 |

**Table S7 – Binary Classification results for cell-type-specific Schizophrenia dataset.**  The table shows five-fold cross validation balanced accuracy (BACC) comparison of DeepGAMI against several machine learning algorithms. Each cell represents average BACC along with standard deviation.

|  | **Microglia** | **Oligodendrocytes** | **Inhibitory Neurons** | **Excitatory Neurons** |
| --- | --- | --- | --- | --- |
| Random Forest | 0.563 ± 0.049 | 0.586 ± 0.036 | 0.576 ± 0.026 | 0.5474 ± 0.047 |
| Naïve Bayes | 0.633 ± 0.05 | 0.634 ± 0.042 | 0.621 ± 0.083 | 0.6469 ± 0.048 |
| MLP | 0.743 ± 0.058 | 0.661 ± 0.023 | 0.715 ± 0.051 | 0.6828 ± 0.051 |
| Varmole | 0.765 ± 0.026 | 0.733 ± 0.053 | 0.741 ± 0.02 | 0.724 ± 0.019 |
| DeepGami Dual | 0.795 ± 0.035 | 0.762 ± 0.053 | 0.755 ± 0.027 | 0.758 ± 0.022 |
| DeepGami Single | 0.784 ± 0.024 | 0.759 ± 0.056 | 0.745 ± 0.036 | 0.746 ± 0.034 |
